# Deformation and energy damage characteristics of granite-concrete composite under uniaxial compression

**DOI:** 10.1371/journal.pone.0316124

**Published:** 2025-03-10

**Authors:** Qingwen Li, Shuhan Gu, Hanjing Li, Wenxia Li, Mengjiao Xu, Yiwei Liu

**Affiliations:** 1 School of Civil and Architectural Engineering, Liaoning University of Technology, Jinzhou, 121001, China; 2 School of Civil Engineering, Shandong Jianzhu University, Jinan, 250101, Shandong, China; Shenyang Jianzhu University, CHINA

## Abstract

To investigate the influence of the fractured rock-concrete interface on the mechanical response of the rock mass and engineering, the mechanical properties and energy evolution of granite-concrete composite specimens with 16 different fracture inclinations were examined through uniaxial compression particle flow simulation. The results show that when the relative area is constant, the larger the fracture dip angle is, the compressive strength of the composite body presents a similar “peak” type change; the dip angle appears to have the maximum value at 60 ^o^ and 90^o^ and the minimum value at 0 ^o^ and 30 ^o^, while the peak elastic modulus presents a “waterfall” type change, and the maximum value appears at 90^o^. The crack types were classified as shear cracks, tensile cracks, secondary shear cracks, secondary tensile cracks, shear-dominated mixed cracks, and tension-dominated mixed cracks. From the crack distribution, it was found that the root cause of crack initiation and propagation was affected by the crack inclination angle. The damage degree increased gradually with the increase of crack inclination angle. When the crack inclination angle was constant, the deterioration degree of the specimen weakened with the increase of relative area s. The elastic energy consumption ratio increases with the shaft deformation, first rapidly and steeply decreasing to the steady inflection point, then slowly increasing to the rapid and steep increase, showing a “fishhook” shape. When the strength failure occurs, the growth speed increases suddenly, and the elastic energy consumption ratio increases suddenly after the *K* peak. This phenomenon can be used as the basis for the occurrence of strength failure and can be used as a qualitative judgment of strength failure.

## 1. Introduction

In practical engineering, rock-concrete composites are often used in building foundations, tunnel supports, dam construction, and so on, while fractured rock-concrete composites exist widely in geological environments. Therefore, the study of mechanical behavior and energy evolution of rock-concrete composites with non-continuous fractures can provide reference for solving practical engineering problems. In recent years, a large number of scholars have carried out the simulation of rock particle flow with prefabricated damage. Xuan et al. [1] analyzed the uniaxial compression damage characteristics of porous rock specimens by PFC and studied how pores affect the crack growth mode, strength, and energy development characteristics of rock. The results show that the existence of pores leads to the strength first decreasing and then increasing. Xie et al. [2] carried out physical uniaxial compression tests on parallel double-joint red sandstone filled with cement mortar under different geometric parameters, deeply analyzed the macroscopic mechanical properties and failure characteristics of red sandstone, and proposed a new method for calculating the stress intensity factor of parallel double-joint joints. Wang et al. [3] developed a physical model of CO2 fracturing to solve the structural stability affected by CO2 fracturing and established a staged damage calculation model based on fractal damage mechanics theory to evaluate rock damage. The results show that the higher the pressure, the more cracks. In addition, Li et al. [4] studied the strength of rock specimens using finite element methods and found that the simple two-dimensional model is very similar to the stress distribution obtained at low stress levels. In addition, at higher stress levels, the simple two-dimensional model produces plastic zones with greater inward displacement than the elastoplastic model. Some scholars have used PFC to carry out uniaxial compression on rock specimens containing cracks [5,6] and found that the peak stress increases with the increase of crack angle.

Scholars at home and abroad have studied the mechanical behavior of rock-concrete composite specimens, and the research results mainly focus on shear resistance [7–10], and there are few research results on other mechanical properties, such as high temperature properties [11,12], freeze-thaw properties [13,14], dynamic properties [15–18], etc. In terms of compressive properties, most scholars [19–22] have carried out fracture tests of rock-concrete interface materials and analyzed the interface crack initiation, crack propagation, and failure process.

At present, scholars at home and abroad have carried out a lot of research on the mechanical properties of intact rock-concrete composite and prefabricated fractured rock and have achieved certain results, but the influence of non-penetrating cracks on the axial compressive properties of rock-concrete composite in two-dimensional plane geometry has not been involved and the role of energy dissipation in granite-concrete composites remains poorly understood and no study has proposed an energy consumption ratio as a failure criterion for granite-concrete composites, which makes this study a pioneering effort in this area. In view of this, PFC is used to construct a numerical analysis model of granite-concrete composite with a reduced size of non-penetrating single fracture, and the numerical model is compared with the experimental results of uniaxial compression in the laboratory. On this basis, the particle flow simulation of granite-concrete composite with prefabricated fracture inclination angle under uniaxial compression is carried out under different factors, by measuring its uniaxial compressive strength, this paper tries to analyze the influence of prefabricated crack inclination on its mechanical properties, crack propagation, and energy evolution under different factors, with a focus on energy consumption ratio as a novel failure criterion, hoping to provide some basic support for the safety state of large-scale geotechnical engineering construction and the stability of engineering structures.

## 2. Microscopic parameter matching and model construction

### 2.1. Indoor tests

The rock material required for the tests were taken from the gneiss granite of the Qirehatal water tunnel, and the strength design grade of the concrete material was C30, with the coordination as shown in Table 1. Complete granite and concrete specimens are cylindrical specimens with size of 100 mm × 50mm (height ×  diameter). For granite-concrete composite specimens, the height ratio of granite to concrete is 1:1, and the interface of the composite is cemented with marble cement. RC-3020 gantry waterjet produced by Foshan City Ruichi Technology Co., Ltd. was used to prefabricate cracks in granite-concrete composite, and WDW-300 microcomputer controlled universal testing machine was used to carry out uniaxial compression test.

**Table 1:** Concrete mix proportion.

Materials	Cement	Mineral powder	Flyash	Sand	Gravel	Admixtures
**Dosage (kg/m**^**3**^)	270	75	45	860	880	8.5

### 2.2. Numerical modelling

**Fig 1:**
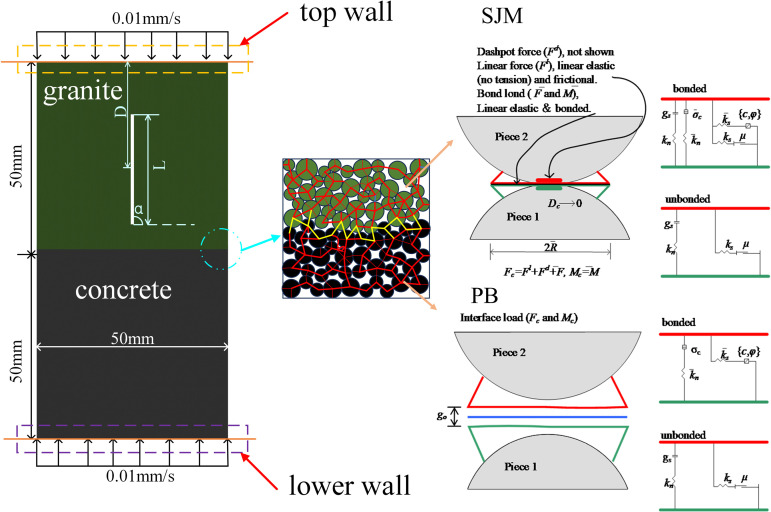
Prefabricated fractured granite-concrete model.


**Fig 2:**
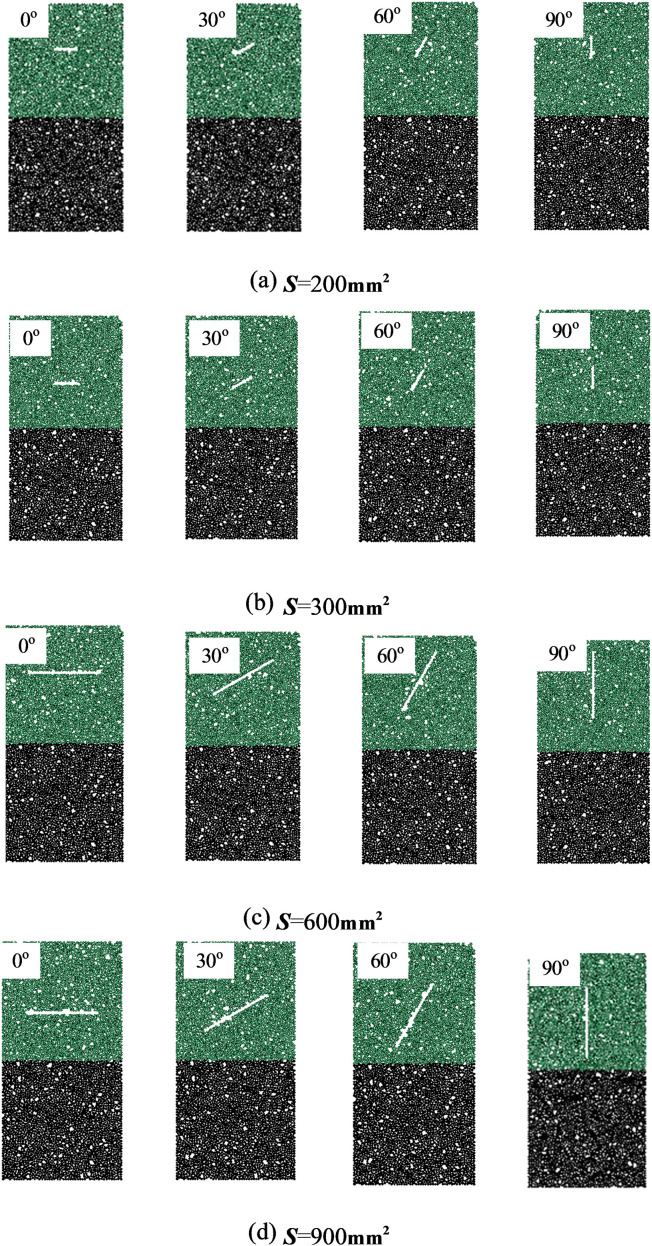
PFC simulation mesoscopic parameters.

In this paper, a uniaxial compression PFC^2D^ model is developed based on the contact types in PFC^2D^ as shown in Fig 1 where the “Linear Parallel Bond Model” (PB) allows the transfer of forces and moments between the particles and shows a very satisfactory response relationship with the rock [23], concrete [24], the “smooth Joint Model” (SJM) has shear and tensile strengths, but does not resist rotation and is commonly used for structural surfaces with frictional properties [25]. Therefore, it was used in this study: the PB model was used inside the two materials and the SJM model was used at the interface. The model dimensions were 50 mm and 100 mm in diameter and height, and 5273 circular particles of different grain sizes were generated in the region, which contained 10248 contacts. Displacement loading method was adopted and loading rate was 0.01 mm/s to simulate uniaxial compression test. The inclination angles of the prefabricated fissures were 0^o^, 30^o^, 60^o^ and 90^o^, the fissure lengths *L* were 10 mm and 30 mm, and *D* of the distances of the fissure centroid from the top were 20 mm and 30 mm respectively. To determine the mechanical behaviour of the prefabricated fissured granite-concrete composite during the loading process corresponding to the assemblage crushing and the energy evolution of the composite, the relationship between the length of the fissure and the distance of the centre of the fissure from the tip is as follows:

S = LD (1)

where *S* is the relative area of the fissure, mm^2^; *L* is the length of the fissure, mm; *D* is the distance of the fissure centre from the tip, mm. The numerical simulation scheme is shown in Fig 2.

### 2.3. Selection and calibration of microscopic parameters

Before the simulation experiment, in order to ensure the accuracy and reasonableness of the simulation results, it is necessary to calibrate the detailed parameters of the specimen model, and the damage mode and energy evolution of the model is affected by the detailed parameters. The mechanical behavior obtained from the indoor uniaxial compression test was used to adjust the model parameters using the ‘parameter optimization’ [26], until the numerical simulation was basically consistent with the indoor test results, as shown in Table 2.

**Table 2:** Parameters PFC simulation microscopic.

Model	Microscopic parameters	Granite	Concrete
**Parallel bonding model**	Particle density/(kg/m^3^)	2790	2360
	Particle friction coefficient	0.3	0.2
	Effective modulus/GPa	17.5	8
	Stiffness ratio	2.53	1.33
	Tensile strength/MPa Bonding strength/MPa	50 150	51 50
	Friction angle/°	30	70
**Rock-concrete interface** **(Smooth joint model)**	Normal stiffness/(N/m)	9 × 10^7^	
	Shear stiffness/(N/m)	45 × 10^7^	
	Frictional coefficient	0.6	
	Cohesion/GPa	20	
	Joint friction angle/°	0.5	

**Fig 3:**
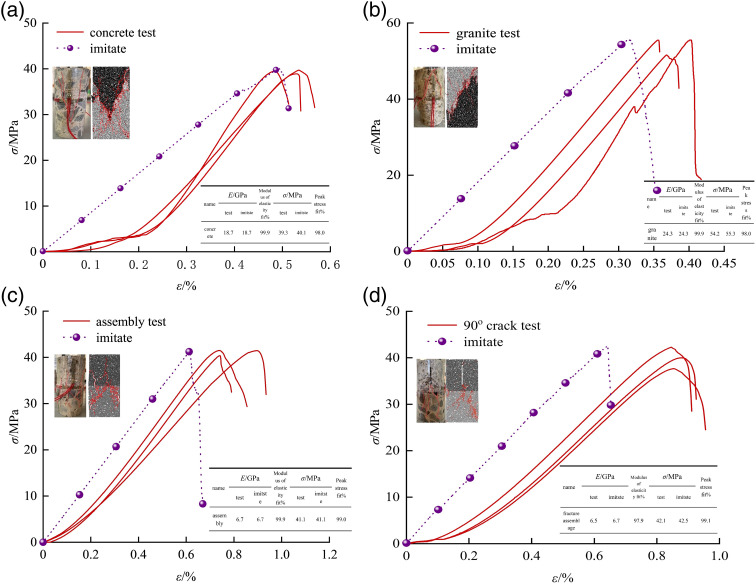
Comparison between the experimental results and the numerical simulation results. (a) concrete (b) granite (c) composite (d) 90^o^crack.

The results of the stress-strain curves of uniaxial compression tests and PFC numerical simulation tests of indoor granite, concrete, intact granite-concrete assemblage and prefabricated fissured granite-concrete assemblage are shown in Fig 3, where the changes of the experimental curves and simulated curves are similar, and the model can accurately reflect their damage characteristics [27]. In the initial stage of the simulated curves, the experimental curves do not show the phenomenon of experimental concave, which is mainly because the main pores will not be compacted when studying the kinematics and mechanical properties of the materials, and therefore the initial compaction and densification phenomenon that occurs in the indoor tests can hardly be reflected in the PFC [28–30].

## 3. Damage mechanical behavior of precast fractured granite-concrete composite

### 3.1. Mechanical characteristics of granite-concrete structure

**Fig 4:**
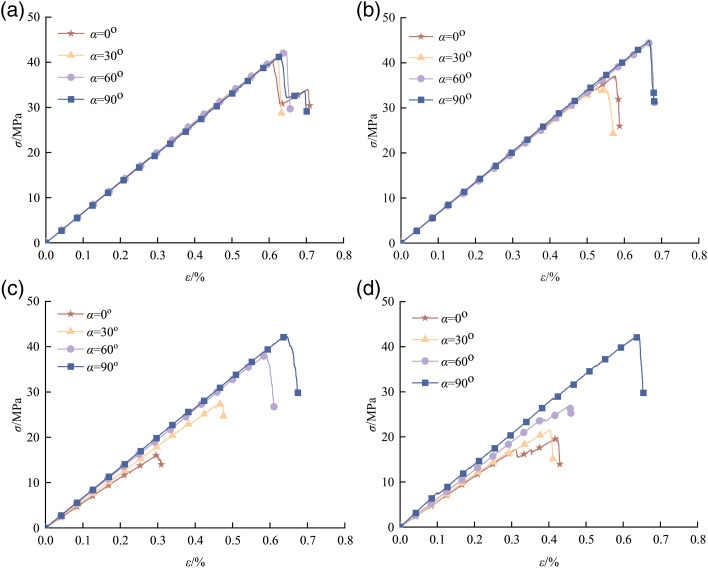
Stress-strain curve of uniaxial compression test of granite-concrete composite. (a) *S* =  200 mm^2^ (b) *S* =  300 mm^2^ (c) *S* =  600 mm^2^ (d) *S* =  900 mm^2^.

Fig 4 shows the stress-strain curve of the uniaxial compression test of granite-concrete composite, with increasing *S*, the stress-strain curves of composite with different crack inclination angles almost overlap in the early stage, and the trend of linear elasticity is rising, which is due to the plastic destruction of granite, resulting in the original fissures and other microstructures inside the granite will be gradually closed by the stress effect, and once loading is started, these microstructures will be gradually compressed. When the stress reaches the peak value, the internal cracks of the specimen will expand rapidly, leading to the destruction of the granite.

As can be seen from Fig 4, the stress-strain curves of the composite with different fracture inclinations show a step-like shape with obvious plastic damage characteristics, and the smaller the relative area is, the more obvious the phenomenon is. With the increase of fracture dip angle, the peak stress and strain of 0^o^ - 60^o^ decrease continuously, from *S* =  200 m^2^ to 900 m^2^, the peak stress of 0^o^ decreases especially obviously, with a decrease of 49.12%.

In the stress-strain curves with different relative areas, the values of peak stress and peak strain are the smallest when the fissure inclination angle is 0^o^, and with the increasing inclination angle, the peak stress and peak strain are also increasing, but with different magnitudes of growth, which is significantly shown in Fig 4d. From Fig 4d, it can be seen that when the fissure inclination angle increases from 0^o^ to 90^o^, the peak strength of the composite gradually increases from 19.704 MPa at 0° by 8.45%, 26.40% and 53.20% respectively. This indicates that the smaller the value of the relative area, the weakening effect on the strength of the composite is significant. Whereas, the larger the relative area, the prefabricated fissure specimens as the inclination angle becomes larger, the peak stress difference increases significantly.

**Fig 5:**
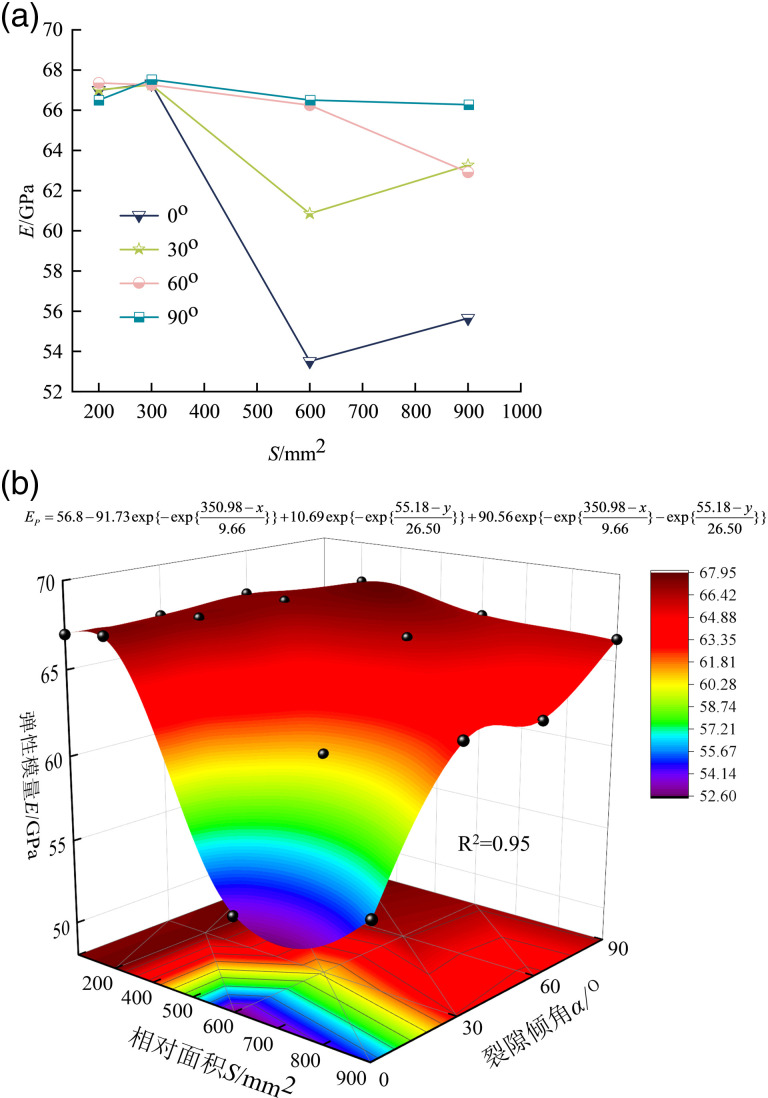
Elastic modulus of precast crack inclination rock-concrete composite with different relative areas. (a) Elastic modulus -relative area relationship (b) Three-dimensional function surfaces.

Fig 5 shows the relationship between the elastic modulus and the relative area and cleavage inclination. From Fig 5a, it can be learnt that the elasticity modulus of the composite generally shows a decreasing trend with the increase of the area.

From Fig 5a, it can be seen that the elastic modulus of the composite with an inclination angle of 0^o^ decreases the most from 200 mm^2^ to 900 mm^2^, from 66.012 GPa to 55.654 GPa, with a decrease of 15.69%. The elastic modulus of the composite exhibits an increasing trend for the fracture inclination from 0^o^ - 90^o^, and *S* =  900 mm^2^, the elastic modulus increases by 11.54%, 12.03%, and 16.03%, respectively, concerning that of the 200 - 600 mm^2^ composite. This is also verified in the trend of the predicted surfaces in Fig 5b. It is also found that the decreasing surface is most pronounced at 0^o^.

### 3.2. Micro-crack propagation characteristics of granite-concrete composite

Through the uniaxial compression test study of rock specimens containing fissure-like rocks, two types of crack distributions were summarised according to the force damage mode, namely tensile and shear cracks.

**Fig 6:**
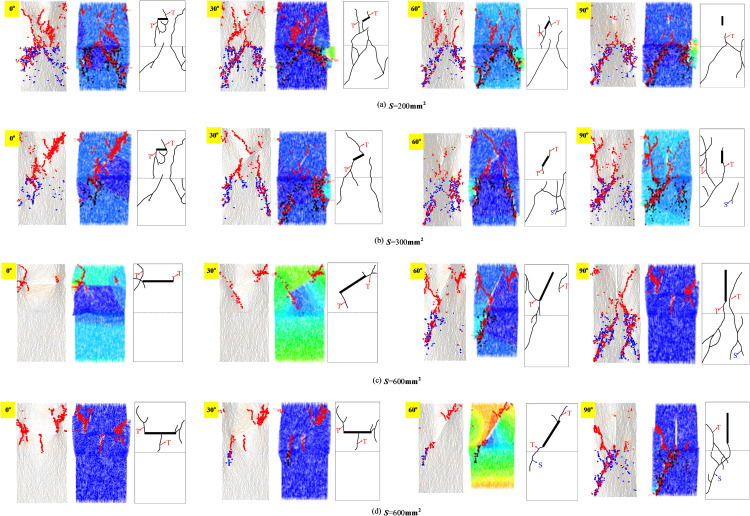
Failure modes of the precast crack inclination rock-concrete composite with different relative areas.

To study the particle motion law of precast fissured granite-concrete composite during uniaxial compression, as shown in Fig 6, the lower side of the interface is the concrete material and the upper side is the granite material, which gives the contact force chain distribution and particle displacement observations in the simulations indicate that, depending on the cracking mechanism and geometry, cracks within the specimen can usually be classified into six types: shear, tensile, secondary shear cracks, secondary tensile cracks, shear-dominated mixed cracks and tensile-dominated mixed cracks. In most cases, tension cracks extend in a relatively stable manner in the direction of the principal stress. Secondary tension cracks arise from the middle of a primary shear crack along a curved path. As the inclination of the prefabricated cracks increased, shear-dominated mixed cracks formed in the concrete material portion, tensile cracks dominated within the granite material and at the interface, and shear cracks dominated within the concrete material, but as the cracks played a role in a variety of modes of crack interaction.

When the relative area *S* is certain, with the increase of crack inclination, the number of cracks in the upper granite material decreases, and the degree of damage gradually decreases, but the opposite is true for the lower concrete material. In addition, the shear cracks do not exist inside the rock, but are fully distributed in the concrete part, which is because after the tensile cracks are generated around the prefabricated cracks in the rock part, the strength of the concrete is smaller than that of the granite, and the shear cracks will follow the tensile cracks’ path to the concrete part. It can be seen that the final damage pattern of the rock-concrete composite is affected by its inclination angle, the damage degree increases gradually with the increase of the crack inclination angle, and the deterioration of the specimen is weakened with the rise of the relative area *S*. This phenomenon is the most significant when the crack inclination angle is at 0^o^, and it can also be seen in the vector diagram of the particle displacement that the specimen is damaged, the directional movement of the particles is obvious, and the upper part of the particles move downward diagonally, the lower part of the particles move in the opposite direction, and a clear shear zone appears, indicating that structural damage occurs within the specimen.

Fig 6 Failure modes of the precast crack inclination rock-concrete composite with different relative areas (The first column is the contact force chain, the second column is the particle displacement; the third column is the crack sketch; where the thickness of the lines of the contact force chain represents the magnitude of the contact force between the individual particles, the grey line indicates the pressure, the orange line indicates the tensile force, the blue indicates the shear crack, and the red indicates the tensile cracks; particle displacement black indicates shear damage cracks and red indicates tension damage cracks; S is the shear damage fissure, T is the tension damage fissure).

## 4. Energy evolution law of prefabricated fractured granite-concrete composite under uniaxial compression

### 4.1. Principles of energy calculation

The deformation and damage process of the specimen under uniaxial compression is essentially an energy conversion process, and the energy evolution of rock-like materials in the compression process until deformation and damage is a dynamic transfer process, which can be decomposed into four stages from the point of view of energy evolution: energy input, energy accumulation, energy dissipation, and energy release [31]. By analysing the energy evolution characteristics of prefabricated fissured rock-concrete composites under uniaxial compression, it is of great significance to reveal the change processes of deformation, breakage and damage of prefabricated fissured rock-concrete composites under uniaxial compression.

It is assumed that during the whole uniaxial compression deformation damage process, the specimen does not exchange heat with the external environment and is a closed system isolated from the outside world. According to the first law of thermodynamics, the equilibrium relationship between energy can be described as:


$$U=U^{e}+U^{d}$$ (2)

where *U* is the total work done by the external force on the rock-like material, J·cm^-3^; *U*^e^ is the elastic energy stored in the material of the composite, J·cm^-3^; *U*^d^ is the dissipated energy during the loading process, J·cm^-3^.

**Fig 7:**
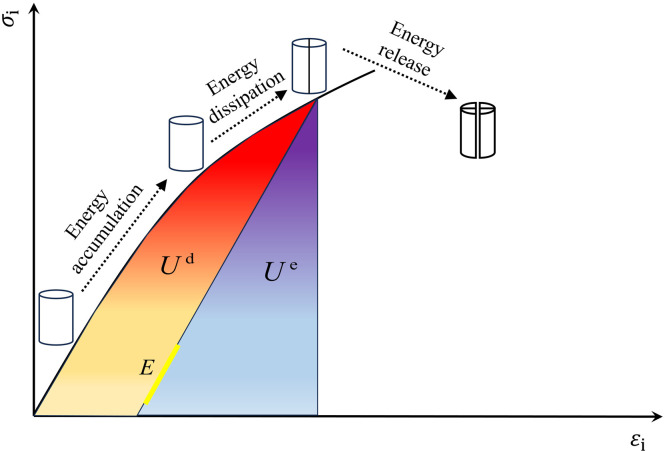
Principle of energy calculation.

Fig 7 shows the principle of energy calculation, which represents the energy conversion of an ideal unit volume for the stress-strain state. The total work is the area under the stress-strain curve, the blue-violet region in the figure is the elastic strain energy, the yellow-red region is the dissipated energy, and the slope is the elastic modulus *E*.

According to the concept of area calculation by definite integral, the total energy is calculated by the formula:


$$U=\sum_{i=1}^{n}\frac{1}{2}(\sigma_{1i}-\sigma_{1-1})(\varepsilon_{1i}-\varepsilon_{1i-1})$$ (3)

The elastic and dissipative energies within the rock are calculated as follows:


$$U^{e}=\frac{\sigma_{1}^{2}}{2E}$$ (4)

$$U^{d}=U-U^{e}$$ (5)

where *E* is the elasticity modulus of the specimen.

### 4.2. Energy evolution laws

**Fig 8:**
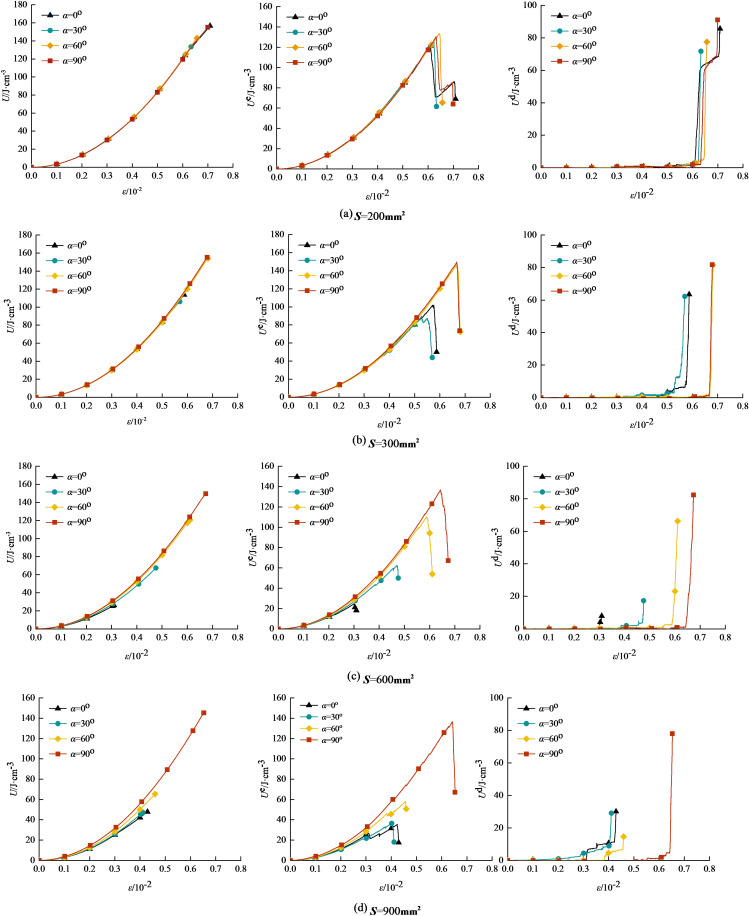
Energy evolution of granite-concrete composite with precast crack Angle under different relative areas.

Based on the simulation results of prefabricated fissure granite-concrete composite under uniaxial compression in section 2.1, using the conversion in the energy calculation method in section 3.1, we can get the uniaxial compression energy evolution law of prefabricated fissure granite-concrete composite with an inclination angle of 0^o^-90^o^ under different relative areas, as shown in Fig 8.

As can be seen from Fig 8, when the relative area gradually increases, the total energy, elastic energy, and dissipation energy are gradually decreasing at the same inclination angle, with the most obvious trend of decreasing from 0^o^ to 60^o^.

When the relative area *S* =  200 mm^2^ - 300 mm^2^, the growth curve of total energy under different fissure inclinations nearly coincides, with the increase of *S*, the energy of fissure inclination 0^o^-90^o^ have different degree of reduction, the elastic energy change rule is with the increase of strain gradually increase, and reach the peak point after the sudden drop; dissipation energy change rule is in the non-peak point when the change is gentle, and reach the peak. The reason is that the fissure granite-concrete composite specimen is destroyed, and the energy stored in it is completely released.

In this paper, the total energy of fissured granite-concrete composite decreases significantly with the increase of *S*. Under the condition of *S* =  200 mm^2^ - 300 mm^2^, the growth curves of the total energy of the fissure inclination angle 0^o^-90^o^ nearly coincide with each other, and both of them are firstly increased gradually with the characteristics of half ‘U’ type, and the total energy decreases most significantly when *S* =  900 mm^2^, the total energy decreases most significantly, compared with *S* =  200 mm^2^, the total energy decreases by 30.50%, 35.34%, 45.73% respectively, which can be seen that the larger the relative area of the specimen, the smaller the fissure inclination, the larger the axial deformation capacity; the elastic energy change curve of granite-concrete composites with different relative areas of crack inclination 0^o^-90^o^ shows an inverted ‘V’ shape, which is very similar to the corresponding stress-strain curve, but the order of the elastic energy size at the peak moment is not consistent with the order of the stress-strain curve, and the most prominent one is *S* =  200 mm^2^, which is the result of the fact that the elasticity modulus is used as a divisor in the calculation method of elasticity energy, and the bigger the elasticity modulus is, the smaller the elasticity energy is.

### 4.3. Total energy at the peak point and its conversion rate

**Fig 9:**
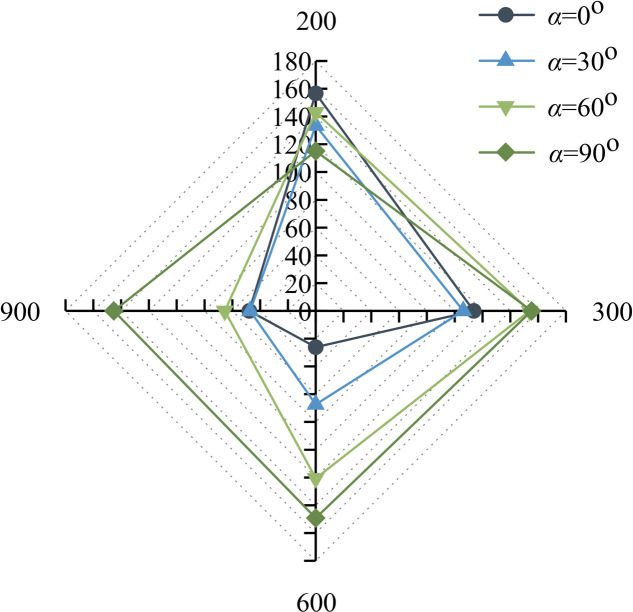
Relative area to total energy at peak point of precast fissure inclined rock-concrete composites.

Fig 9 shows the total peak point energy of granite-concrete composite with prefabricated fissure inclination 0^o^-90^o^ under different relative areas, the total peak point energy of granite-concrete composite with prefabricated fissure inclination decreases with the increase of relative area. When *α* =  0^o^, with the increase of relative area, the peak point total energy firstly decreases with a slightly smaller magnitude of 27.45% and then decreases steeply with a larger magnitude of 75.65%, and then increases rapidly with a smaller magnitude of 45.33%, the magnitude of which is jagged, and the maximum peak point total energy is obtained when *S* = 200mm^2^ and the minimum peak point energy is obtained when relative area is the largest, and the fissure inclination angle of 30^o^-60^o^, the total energy of the peak points is reduced with the increase of relative area. When the relative area is 30^o^-60^o^, with the increase of relative area, the total energy of the peak point is similar to the trend when the fissure inclination is 0^o^; when *α* =  90^o^, with the increase of relative area, the total energy of the peak point is firstly increased rapidly with a large magnitude of 34.91%, then decreased with a small decrease of 3.61%, and then continued to be decreased with a very small decrease of 2.67%, the amplitude is in the shape of ‘へ’, the maximum value of the total energy at the peak point is obtained when *S* =  300mm^2^, and the minimum value of the total energy at the peak point is obtained at the maximum relative area. It can be seen that the peak point total energy of prefabricated fissure inclination granite-concrete composite was significantly reduced, and the significance of the effect of fissure inclination on the peak point total energy of granite-concrete composite was better than that of the relative area. Using this energy-based failure criterion, it can be used to predict practical applications of granite-concrete composites in construction and mining.

**Fig 10:**
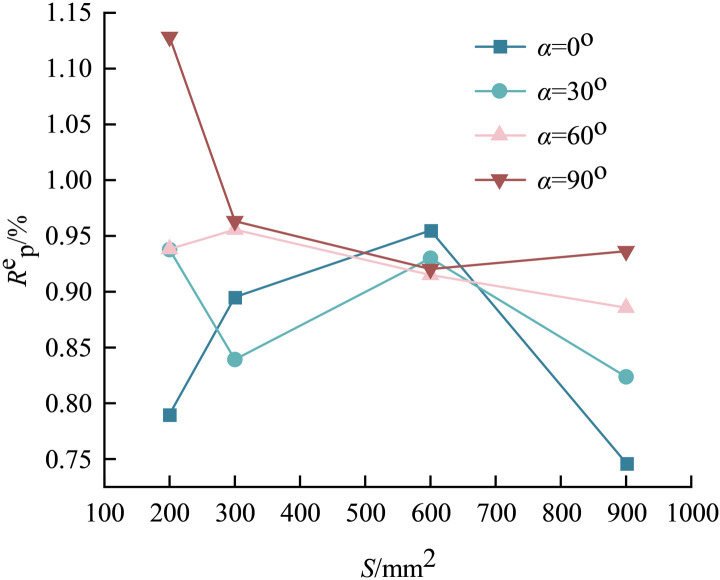
Conversion rate of elastic energy of precast fissure inclined rock-concrete composite by relative area.

To describe the energy conversion of precast fissure inclined granite-concrete composites under uniaxial compression, the elastic energy conversion rate at peak stress, defined as the ratio of elastic energy to total energy, in %, was investigated. The elastic energy conversion rates of precast fissure-inclined granite-concrete composites with different relative areas are given in Fig 10, and the elastic energy conversion rates generally show a decreasing trend with the increase of relative areas. Future work on this approach could explore the effects of environmental factors such as temperature and humidity on the failure characteristics of granite-concrete composites and extend this approach to other composites.

From 200 mm^2^-900 mm^2^, the change of inclination angle 0^o^ and 90^o^ is the largest, and the prefabricated fissure inclination granite-concrete assemblage at *α*=0° varied in the interval of 0.79 ~ 0.95,with the increase of the relative area, it initially increases by 12% and then increases by 6.7%, and then decreases by 21.02%, which looks like a ‘Fishhook’ shape, when the relative area is the largest, $R_{\text{p}}^{\text{e}}$ is the smallest, the relative area of 600 mm^2^ is the largest; prefabricated fissure inclination granite-concrete assemblage at *α* =  0^o^ varies in the interval from 0.936 to 1.128, with the increase in the relative area, $R_{\text{p}}^{\text{e}}$ first decreases sharply 17.13% and then 4.67% of the amplitude of the slow decrease, and then 1.71% of the smaller amplitude of the slow increase, similar to the “spur” shape, $R_{\text{p}}^{\text{e}}$ is maximum when the relative area is 600 mm^2^. It can be seen that both the relative area and the inclination angle of the fissure have an effect on the elastic energy conversion rate of the granite-concrete composite, but the influence of the inclination angle is more significant than that of the relative area, and the higher the elastic energy conversion rate is, the larger the proportion of the elastic energy is, and the stronger the impact of the test specimen is when it destroys.

**Fig 11:**
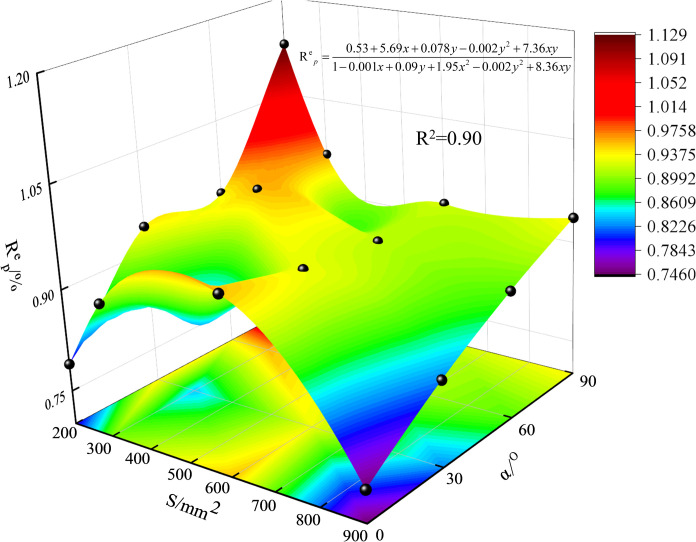
Elastic energy conversion surface of precast crack angle rock-concrete composite with different relative areas.

Combined with the elastic energy conversion rate of prefabricated granite-concrete composites with different relative areas, as shown in Fig 11, the predicted surface trend also verifies the aforementioned conclusion that ‘the effect of fissure inclination on the elastic energy conversion rate is more significant than that of relative area’.

### 4.4. A preliminary study on the energy criterion for the instability of granite-concrete composite at fissure inclinations

The strength failure of rock under high stress and strain does not necessarily lead to the overall destruction of the rock mass [32–35]. Assuming that work is done on an ideal homogeneous material to produce any deformation, the total volume of this material does not change, the total energy absorbed is all converted into elastic energy inside the material, and there is no plastic deformation in the deformation process, and *U*^d^ =  0. However, there are processes of plastic deformation, cracks producing extension and penetration, and internal friction in rock and concrete materials. The whole loading process can be understood as a transformation process between steady state and unstable state: steady state (intact specimen) at the beginning of loading, developing into unstable state (main crack penetration) after loading for some time, and then back to the steady state (multiple fragments after the destruction of the whole specimen, and at this time the strength is the residual strength). This process can be regarded as the absorption, transformation and release of energy, so the sudden change of energy can be used as a strength failure criterion. The elastic energy consumption ratio *K* is defined as the ratio of dissipated energy to elastic energy in the total energy input to the material to characterise in real time the state of energy dissipation and storage during the whole process of force deformation and destruction of the specimen, which is expressed by the following equation:


$$K=\frac{U^{\text{d}}}{U^{\text{e}}}$$ (6)

**Fig 12:**
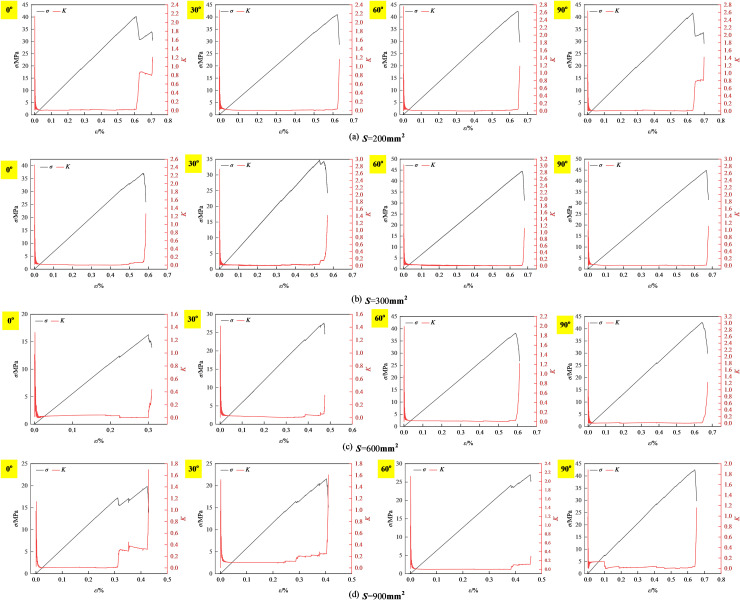
Elastic energy consumption rate-stress-strain curve of precast crack inclination rock-concrete ccomposite with different relative areas.

Based on the elastic properties and dissipated energy data in Fig 8 and converted using Eq. (6), the elastic-energy ratio-stress-strain curves of prefabricated fissure inclination 0°-90° granite-concrete composites with different relative areas are given, as shown in Fig 12.

The elastic energy consumption ratio-stress-strain curves of prefabricated fissure inclination granite-concrete composite under different relative areas are shown in Fig 12.

Fig 12 shows that the elastic energy consumption ratio-stress-strain curves of prefabricated granite-concrete composites with different relative areas are similar. In the initial loading stage, the elastic energy consumption ratio increases sharply at different fissure inclination angles, and then decays rapidly, and the decay rate increases with the increase of *S* =  300 mm^2^, and the dissipated energy ratio reaches the maximum value of *K*_max_ at the initial stage, which is 2.46, 2.72, 2.92, and 2.82, respectively, due to the compaction of the fissure. The initial stage is due to the high percentage of dissipated energy caused by the fracture compaction; subsequently, the elastic energy consumption ratio gradually decreases to 0 <  *K* <  1, and the closure of primary fissures and other initial damages leads to energy dissipation at this stage, and most of the primary fissures are closed, the elastic strain energy is rapidly stored, accompanied by a small amount of microcracks; finally, when *K* >  1, the growth rate of elastic energy dissipation ratio increases and shows a steep increase, at this time, the microcracks inside the rock-concrete ccomposite are gradually connected to form a macroscopic fissure, and the previously accumulated elastic energy is rapidly released, the dissipation energy increases sharply, and the composite destabilises and destroys as the loading proceeds.

The elastic energy consumption ratio *K*, defined as the ratio of *U*_d_ to *U*_e_ [36], can be used as an amplified energy signal in the event of strength failure of prefabricated fissured granite-concrete composites, and its abrupt change can be used as a strength failure criterion. It provides some references for different working faces or engineering stability.

## 5. Conclusion

In actual engineering, the geological body and engineering body often bear the action together, and the defects such as microfractures, cracks and fissures existing inside the natural rock materials will reduce the strength of the rock body and the stability of the engineering body. Based on the results of indoor tests, uniaxial compression numerical tests on prefabricated fissured rock-concrete composite with different relative areas are carried out by the PFC particle flow procedure to investigate the mechanical behaviours of prefabricated fissured rock-concrete composite with different relative areas, analyse the particle contact force chain and displacement field of the damaged specimens, and study the energy evolution of the composite under the influence of different relative areas and fissure inclinations. The main conclusions are as follows:

1. With the increase of relative area and the change of fracture inclination angle, the compressive strength of precast fractured granite-concrete composite decreases gradually. This indicates that the prefabricated fissure has a significant weakening effect on the strength of the composite; while the larger the fissure length, the prefabricated fissure specimen shows a significant increase in the peak stress difference as the inclination angle becomes larger.
2. Under the influence of compressive and tensile stress concentrations, the lower surface of the prefabricated slit was the first to develop tensile cracks. The concentration of tensile stresses at the tip of the expanding crack indicates that contact failure and slip are most likely to occur in the strong tensile zone. The root cause of crack initiation and extension is the change and transfer of the stress field. Shear cracks will follow the path of tensile cracks and extend towards the concrete part, so shear cracks are all distributed in the concrete part and do not exist in the rock part. The direction of particle movement in the lower part of the crack is opposite to that of the upper part, and a clear shear zone appears.
3. At *α* =  0°, the total energy at the peak initially decreases with increasing relative area, then continuously increases beyond 145.253 J·com^-3^; when *α*=90°, with the increase of relative area, the total energy at the peak point increases and then decreases continuously. The effect of the fissure inclination on the elastic energy conversion rate is more significant than that of the relative area, and the smaller the relative area, the larger the fissure inclination, and the larger the conversion rate, which reveals the evolution law of the elastic energy conversion rate of the rock-concrete composites with fissure inclination from 0°-90° under different relative areas, and establishes a surface that takes into account the relationship between the relative area and the fissure inclination.
4. With the increase of axial deformation, the elastic energy consumption ratio of the precast fractured granite-concrete composite body decreases rapidly at first, then gradually decreases to the inflection point, and then increases gradually to a rapid and steep increase. The elastic energy consumption ratio near the peak value appears to be a continuous, step-like, abrupt change, which indicates that fractures are developing rapidly. This step-like abrupt change phenomenon can be used as the judgment basis for instability and failure of a precast fractured granite-concrete composite body.

## Supporting information

S1 Dataset Clear pictures in the paper. (ZIP)

S1 Table Clear tables (ZIP)

S1 Minimal Data Set Data from the picture table in the paper. (ZIP)
