# Supplementary figures and images for: Deformation and energy damage characteristics of granite-concrete composite under uniaxial compression
Source: PLoS One. 2025 Mar 10;20(3):e0316124. doi: 10.1371/journal.pone.0316124 (PMC11892862; doi:10.1371/journal.pone.0316124)

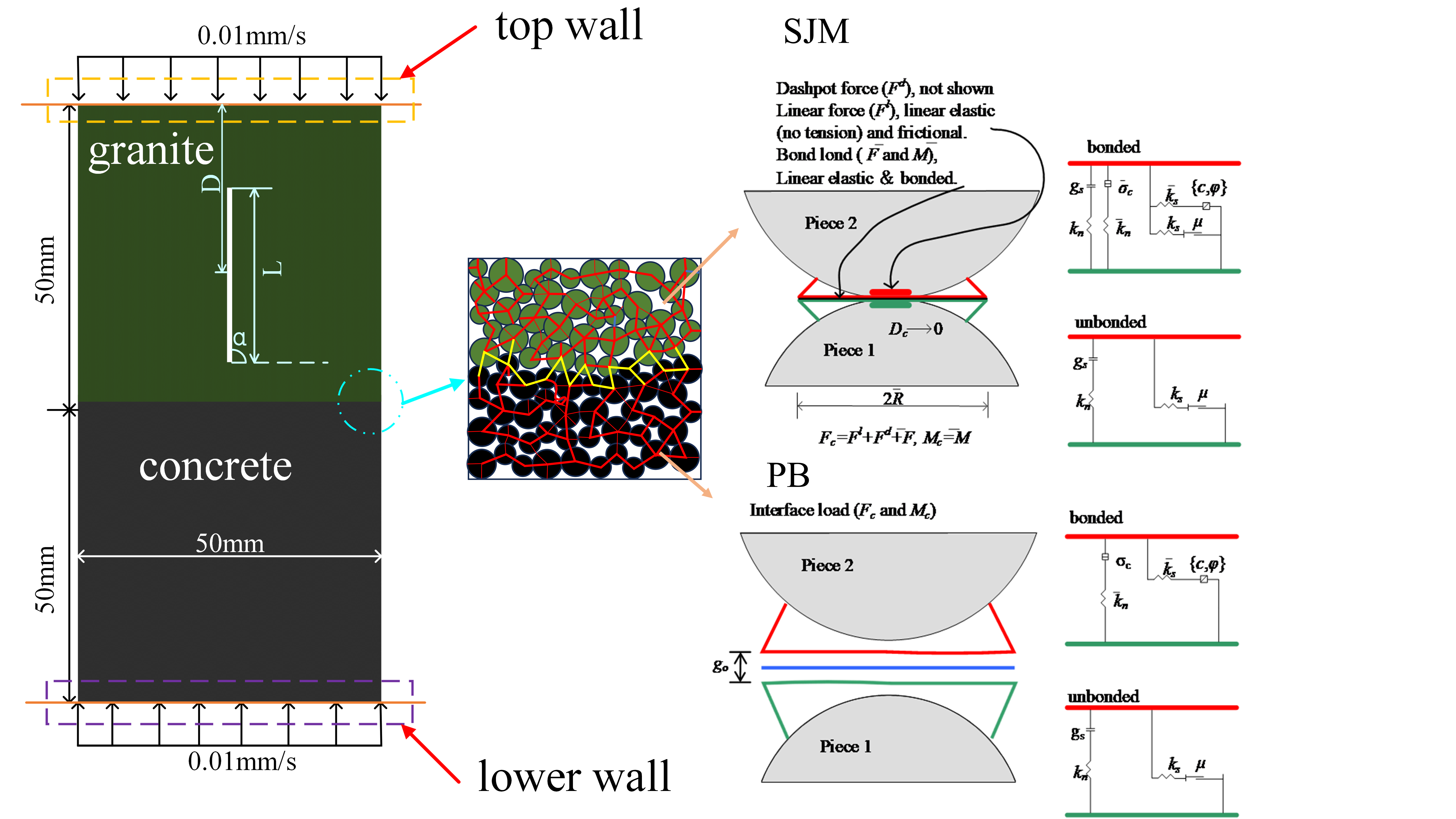

Supplement: S1 Dataset — (ZIP) [file pone.0316124.s001.zip › S1 Dataset/Fig 1. Prefabricated fractured granite-concrete model.tif]

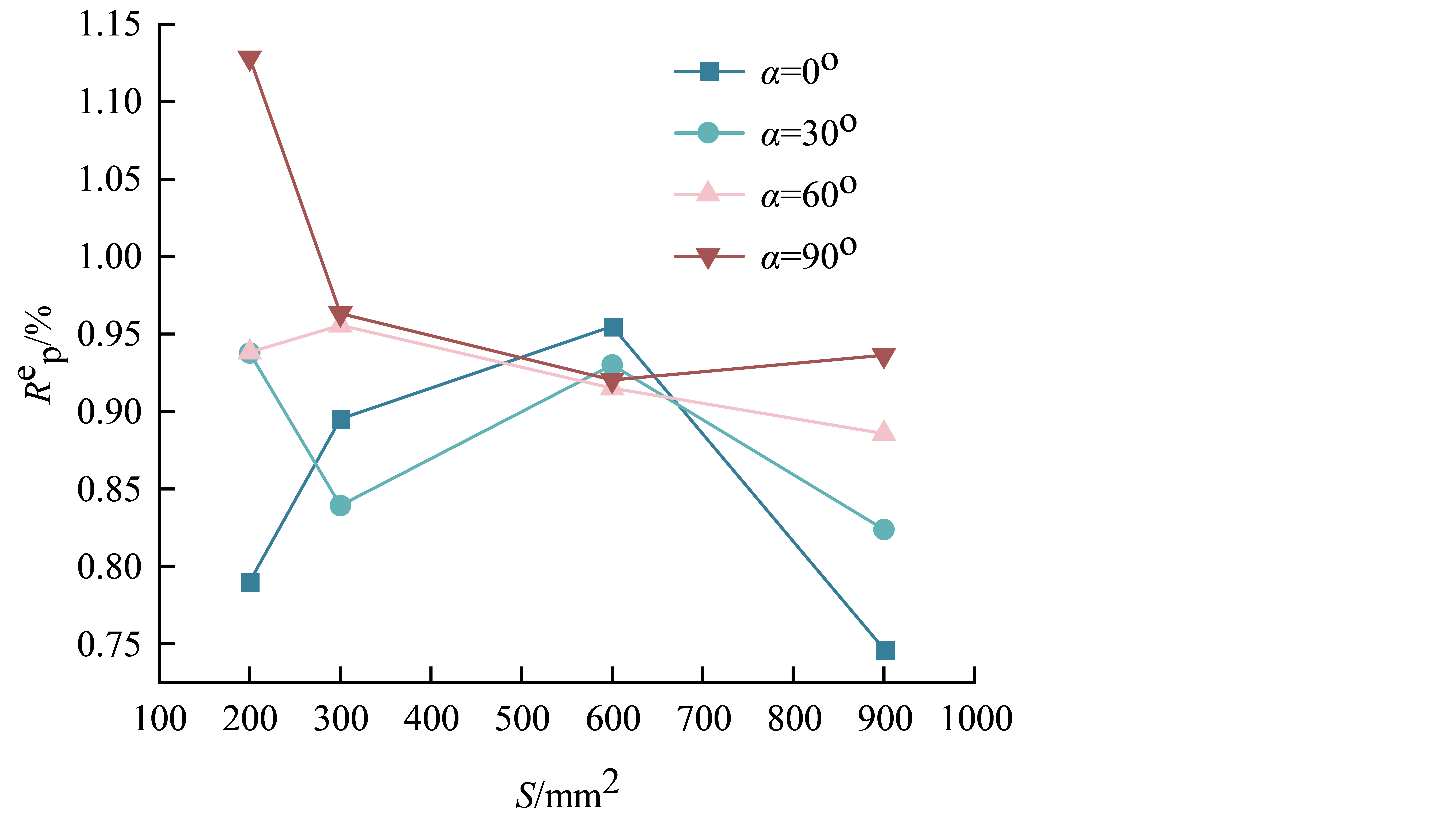

Supplement: S1 Dataset — (ZIP) [file pone.0316124.s001.zip › S1 Dataset/Fig 10. Conversion rate of elastic energy of precast fissure inclined rock-concrete composite by relative area..tif]

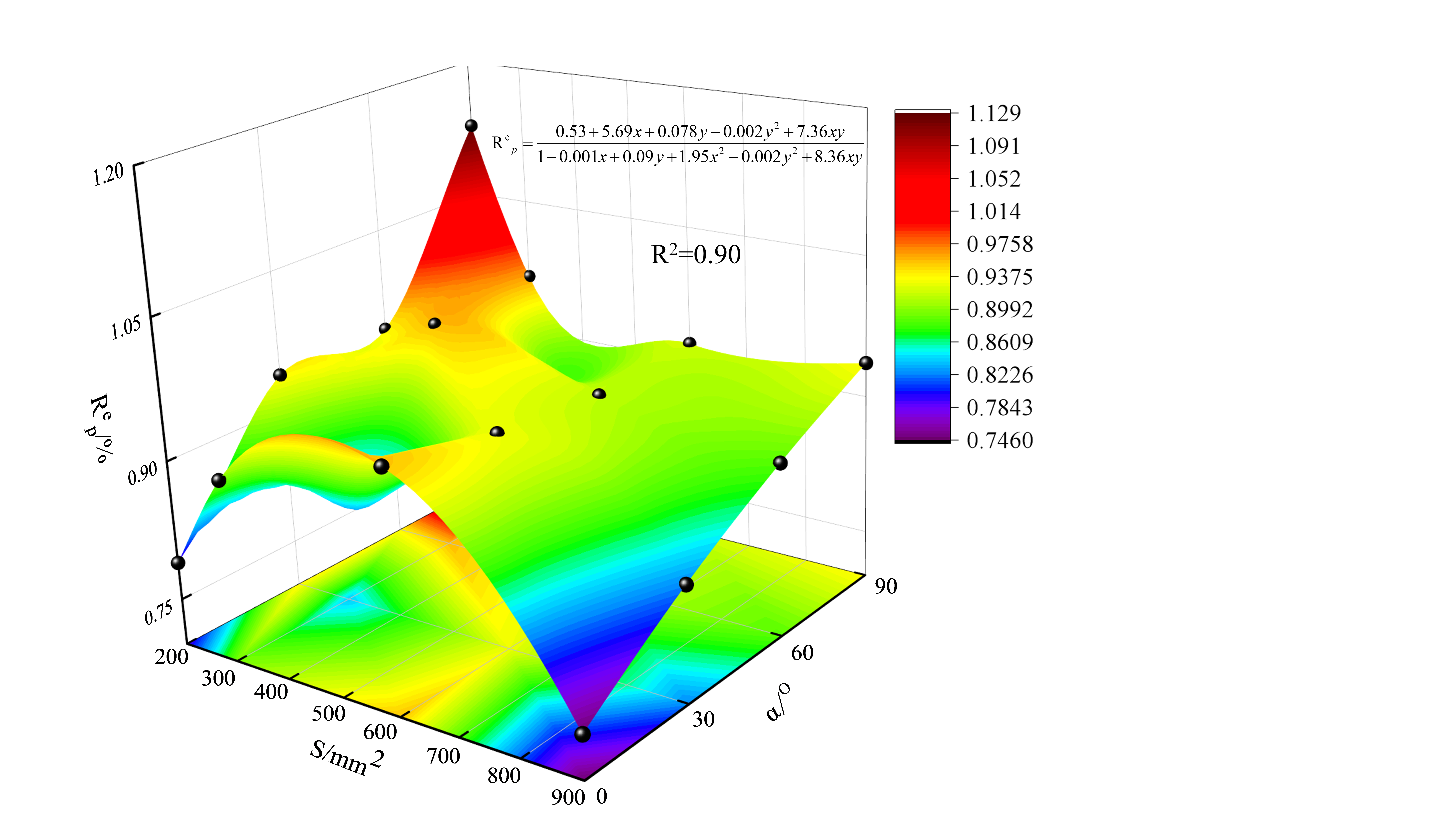

Supplement: S1 Dataset — (ZIP) [file pone.0316124.s001.zip › S1 Dataset/Fig 11. Elastic energy conversion surface of precast crack Angle rock-concrete composite with different relative areas.tif]

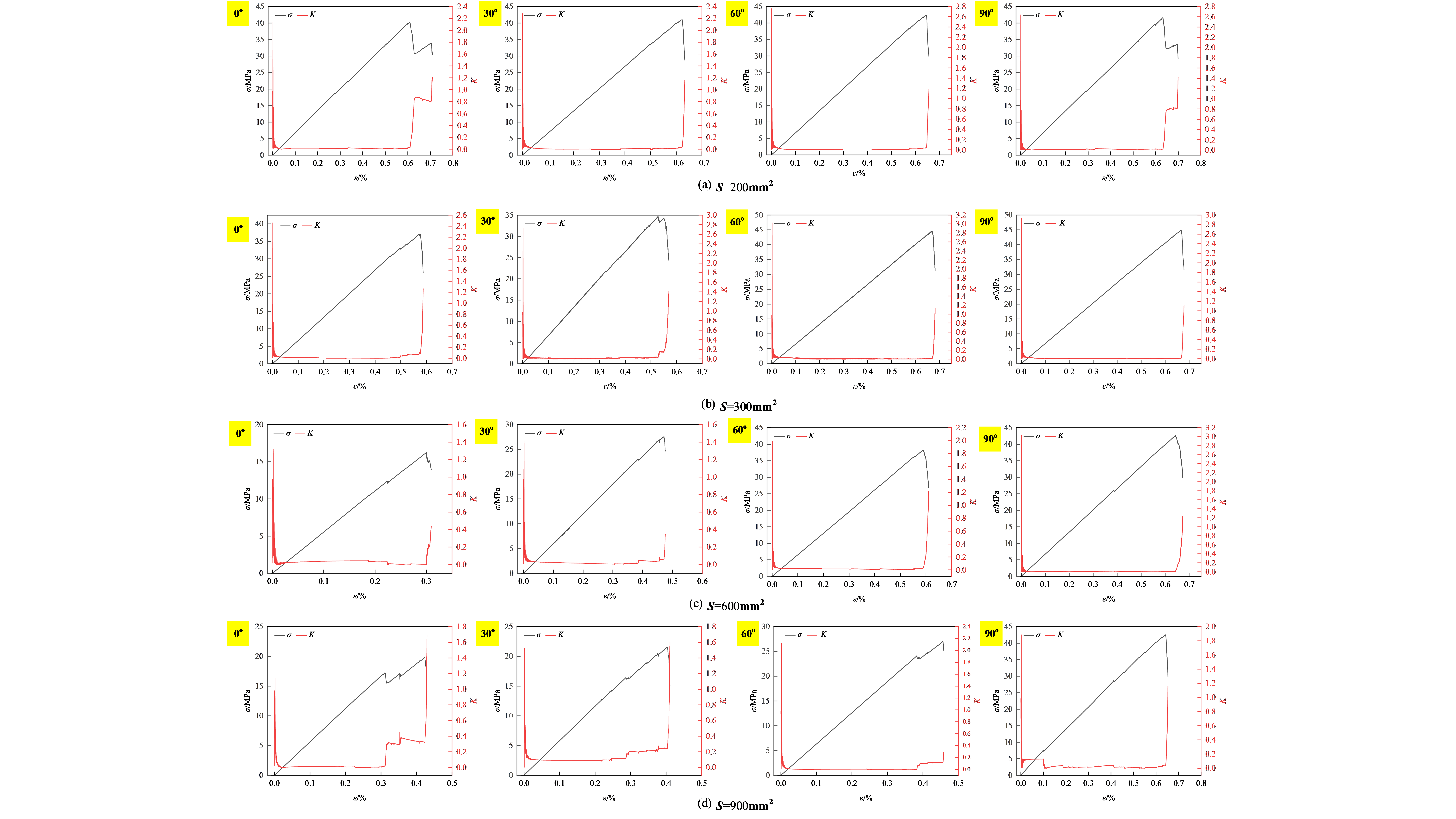

Supplement: S1 Dataset — (ZIP) [file pone.0316124.s001.zip › S1 Dataset/Fig 12. Elastic energy consumption rate-stress-strain curve of precast crack inclination rock-concrete ccomposite with different relative areas..tif]

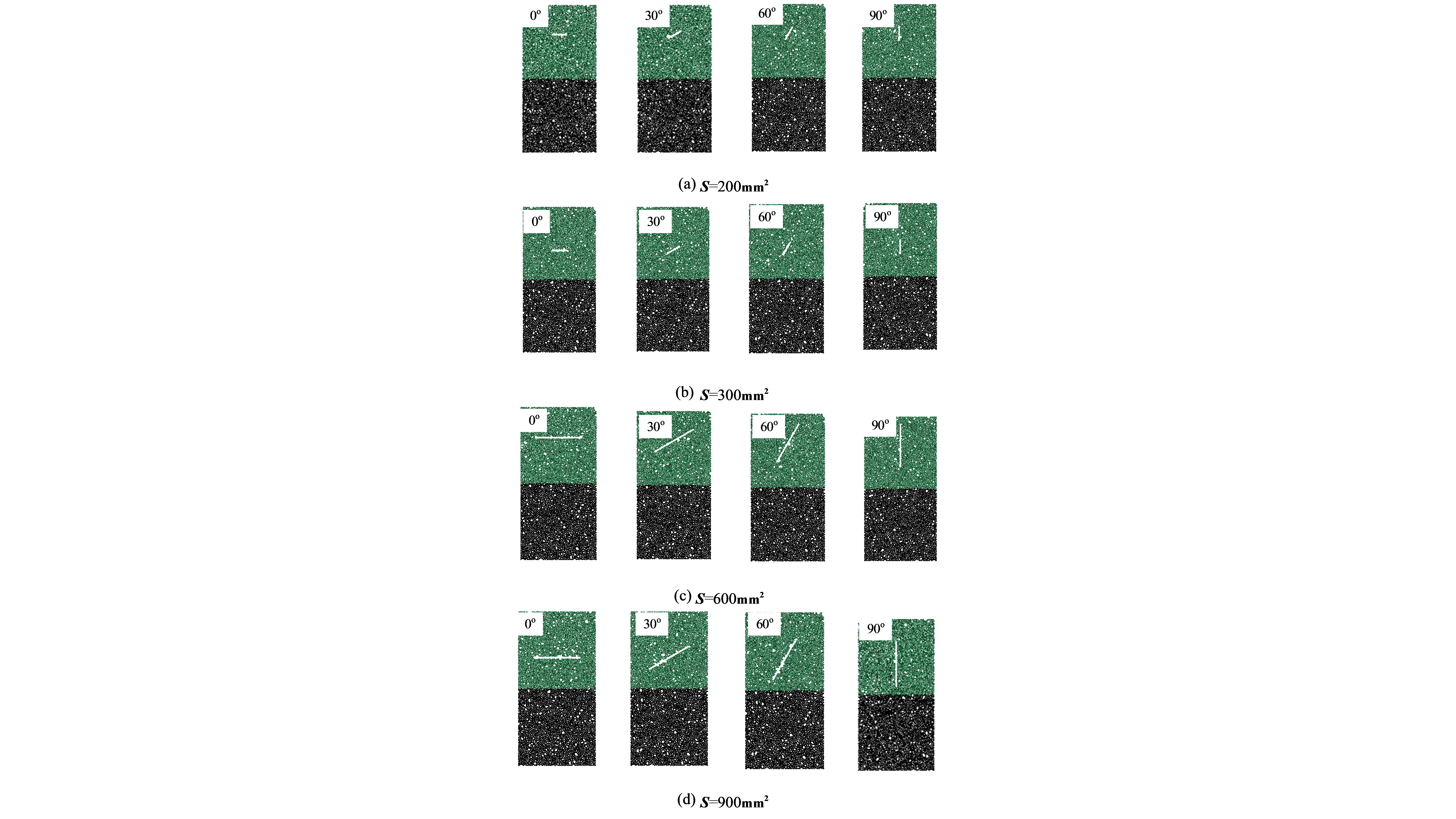

Supplement: S1 Dataset — (ZIP) [file pone.0316124.s001.zip › S1 Dataset/Fig 2. PFC Simulation Mesoscopic Parameters.able 3. Parameters PFC simulation microscopic..tif]

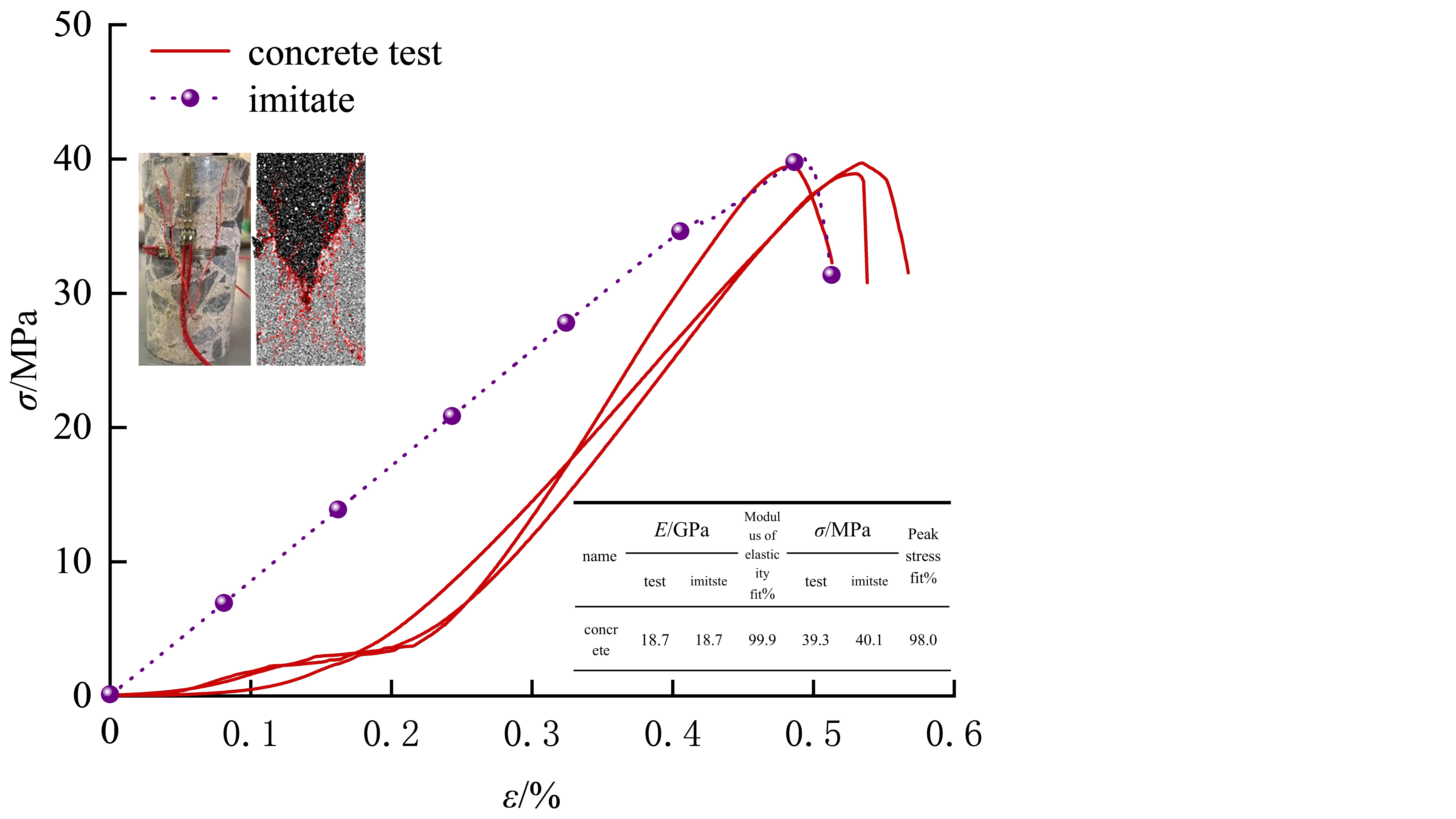

Supplement: S1 Dataset — (ZIP) [file pone.0316124.s001.zip › S1 Dataset/Fig 3. (a) Comparison between the experimental results and the numerical simulation results.tif]

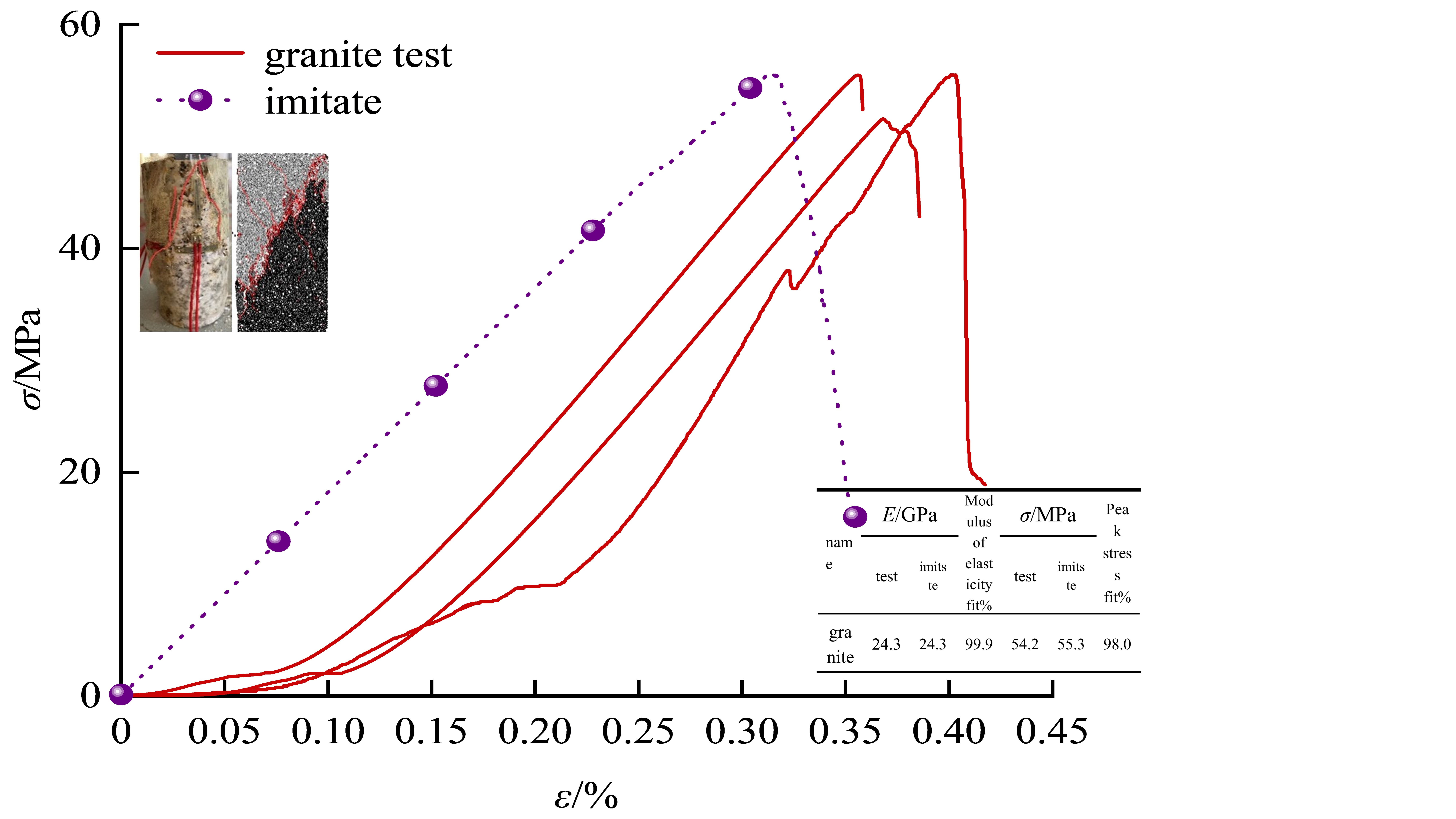

Supplement: S1 Dataset — (ZIP) [file pone.0316124.s001.zip › S1 Dataset/Fig 3. (b) Comparison between the experimental results and the numerical simulation results.tif]

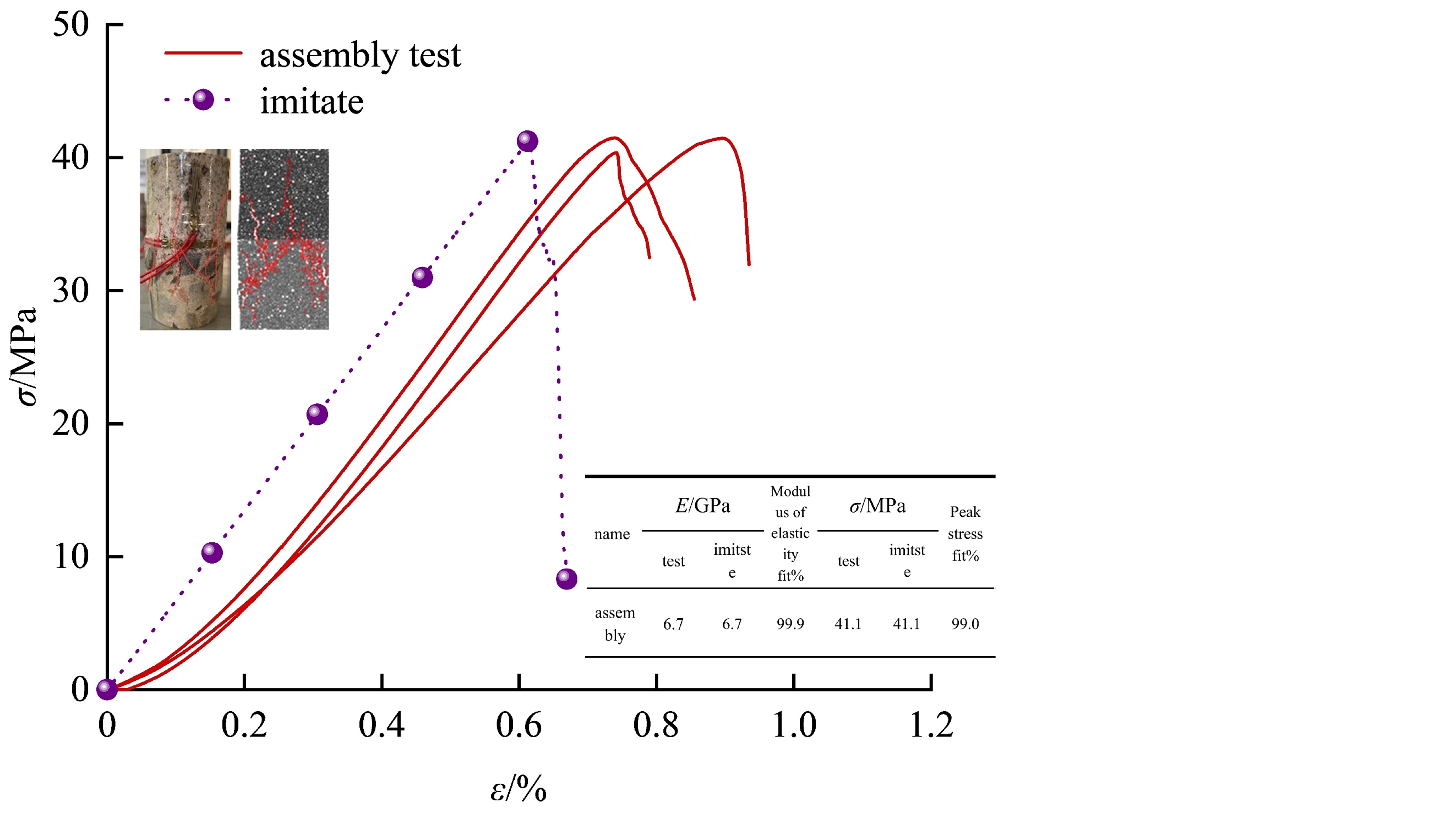

Supplement: S1 Dataset — (ZIP) [file pone.0316124.s001.zip › S1 Dataset/Fig 3. (c) Comparison between the experimental results and the numerical simulation results.tif]

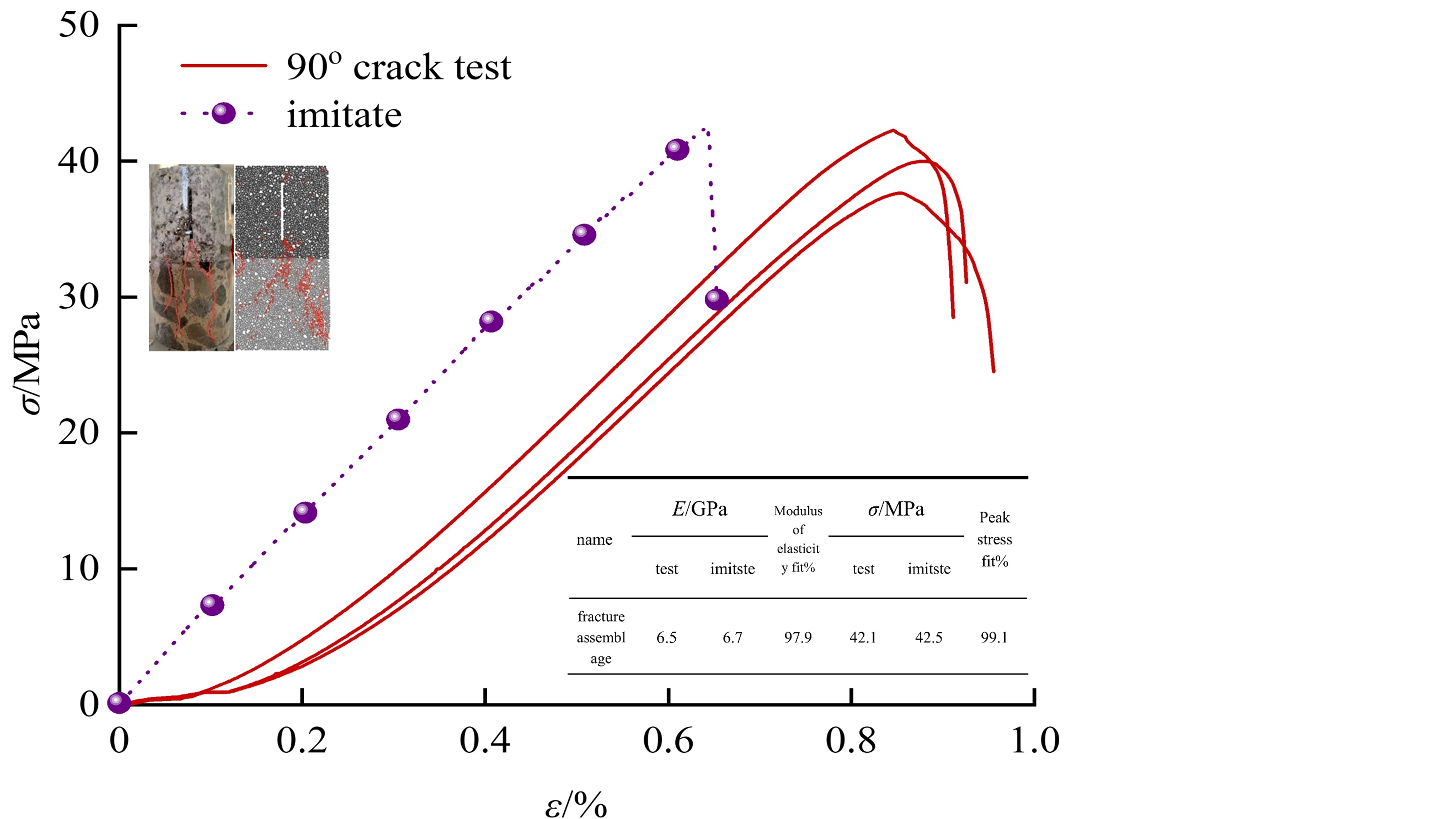

Supplement: S1 Dataset — (ZIP) [file pone.0316124.s001.zip › S1 Dataset/Fig 3. (d) Comparison between the experimental results and the numerical simulation results.tif]

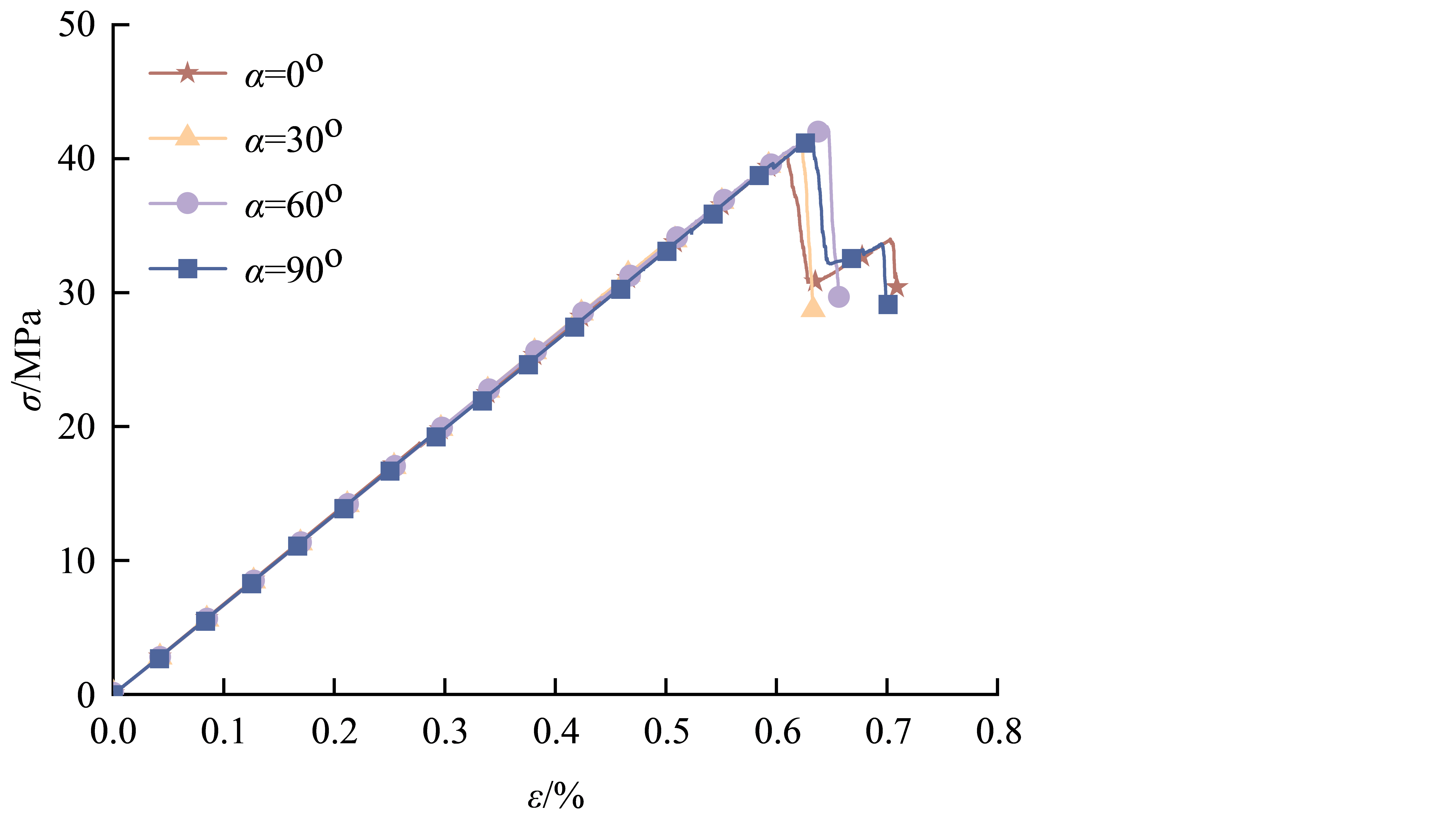

Supplement: S1 Dataset — (ZIP) [file pone.0316124.s001.zip › S1 Dataset/Fig 4.(a) Stress-strain curve of uniaxial compression test of granite-concrete composit.tif]

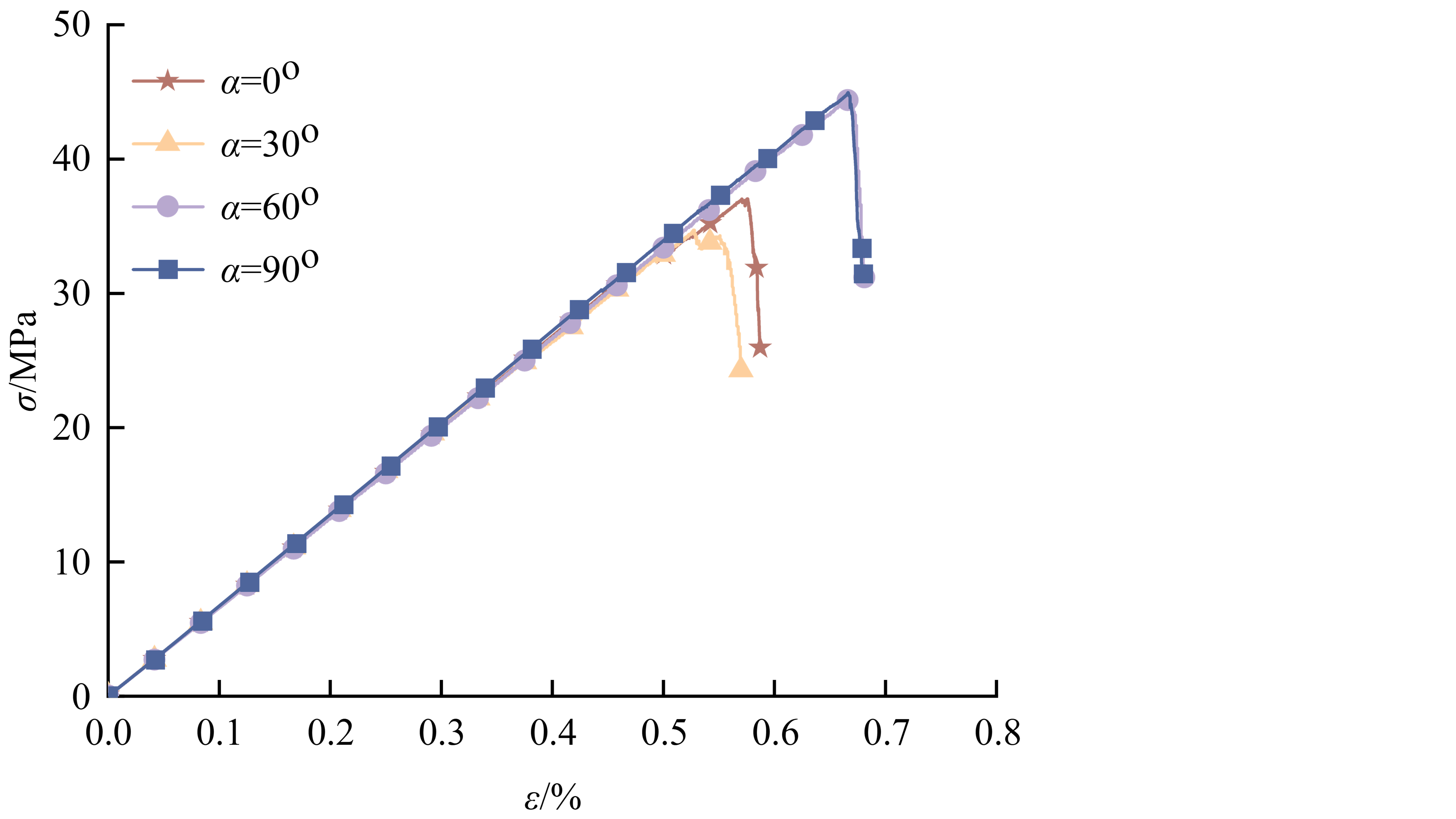

Supplement: S1 Dataset — (ZIP) [file pone.0316124.s001.zip › S1 Dataset/Fig 4.(b) Stress-strain curve of uniaxial compression test of granite-concrete composit.tif]

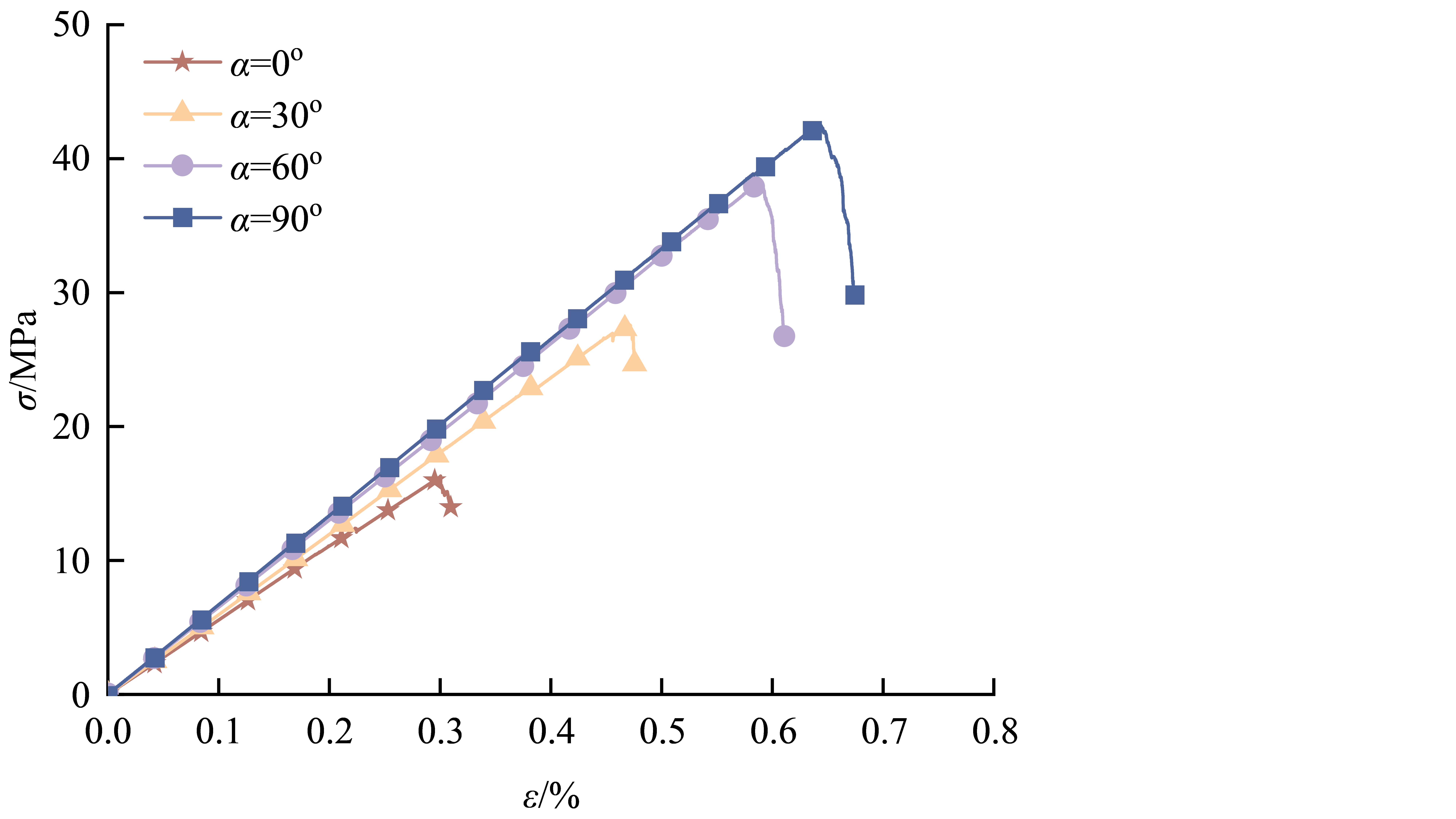

Supplement: S1 Dataset — (ZIP) [file pone.0316124.s001.zip › S1 Dataset/Fig 4.(c) Stress-strain curve of uniaxial compression test of granite-concrete composit.tif]

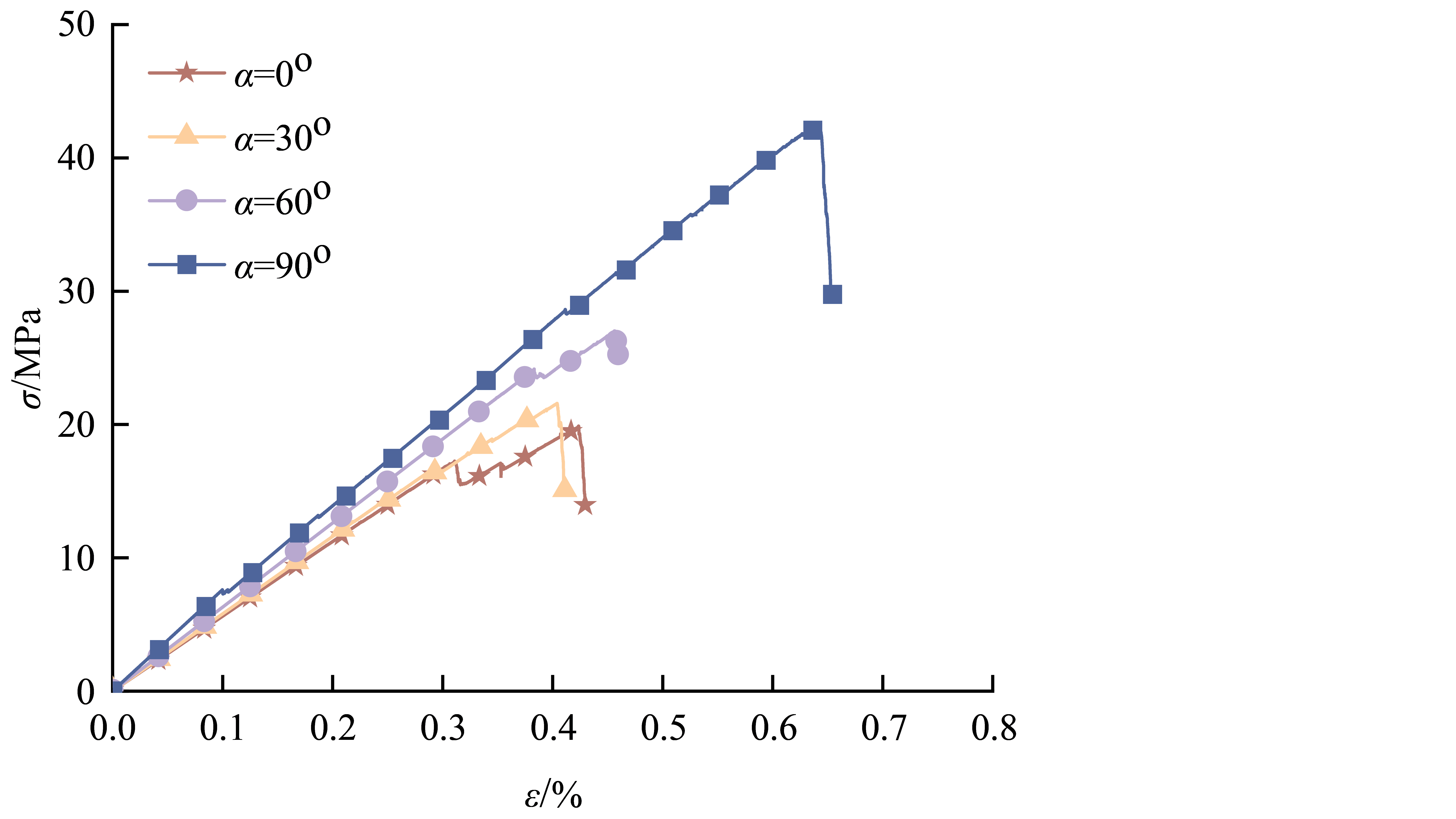

Supplement: S1 Dataset — (ZIP) [file pone.0316124.s001.zip › S1 Dataset/Fig 4.(d) Stress-strain curve of uniaxial compression test of granite-concrete composit.tif]

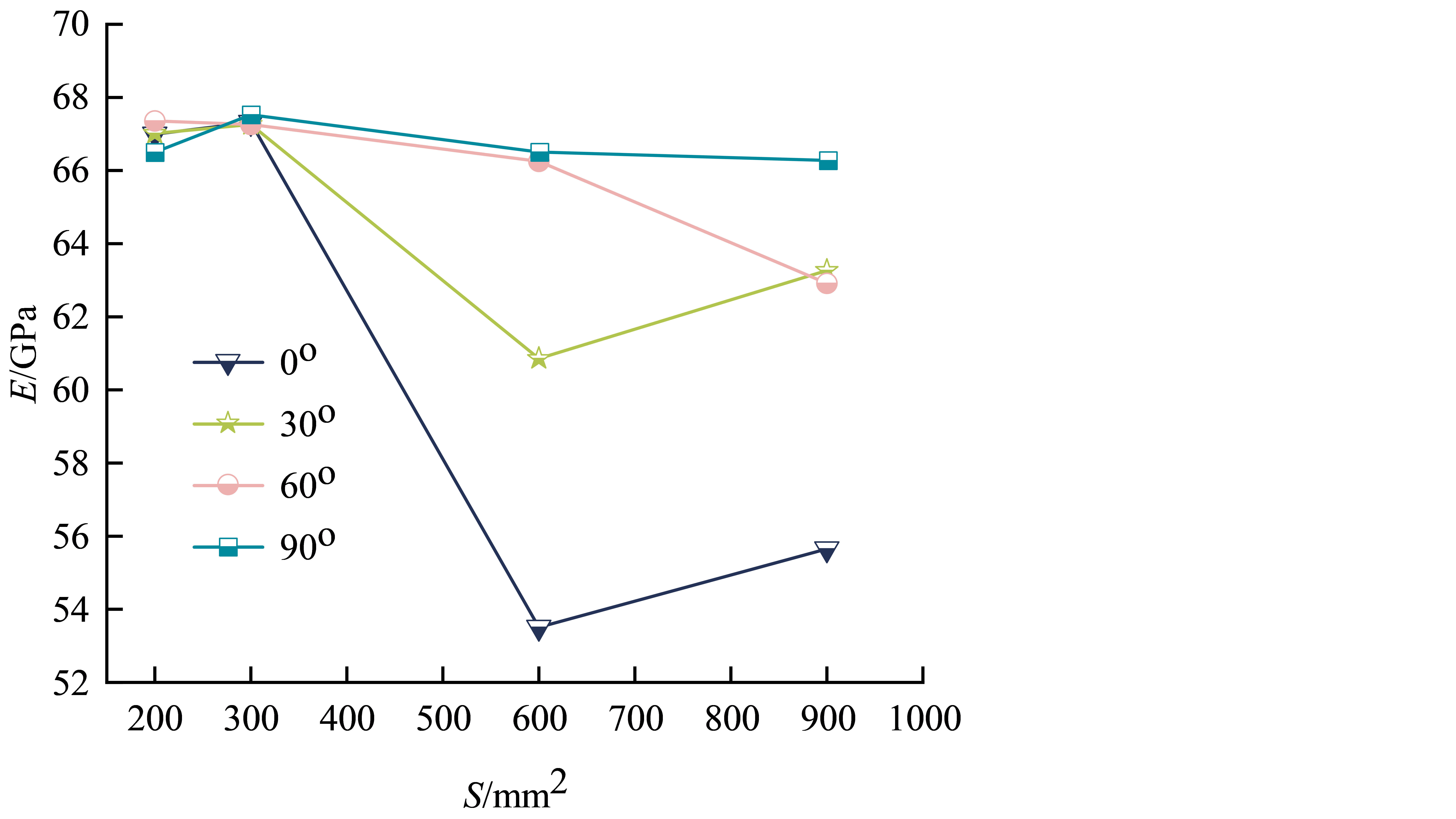

Supplement: S1 Dataset — (ZIP) [file pone.0316124.s001.zip › S1 Dataset/Fig 5. (a) Elastic modulus of precast crack inclination rock-concrete composite with different relative areas.tif]

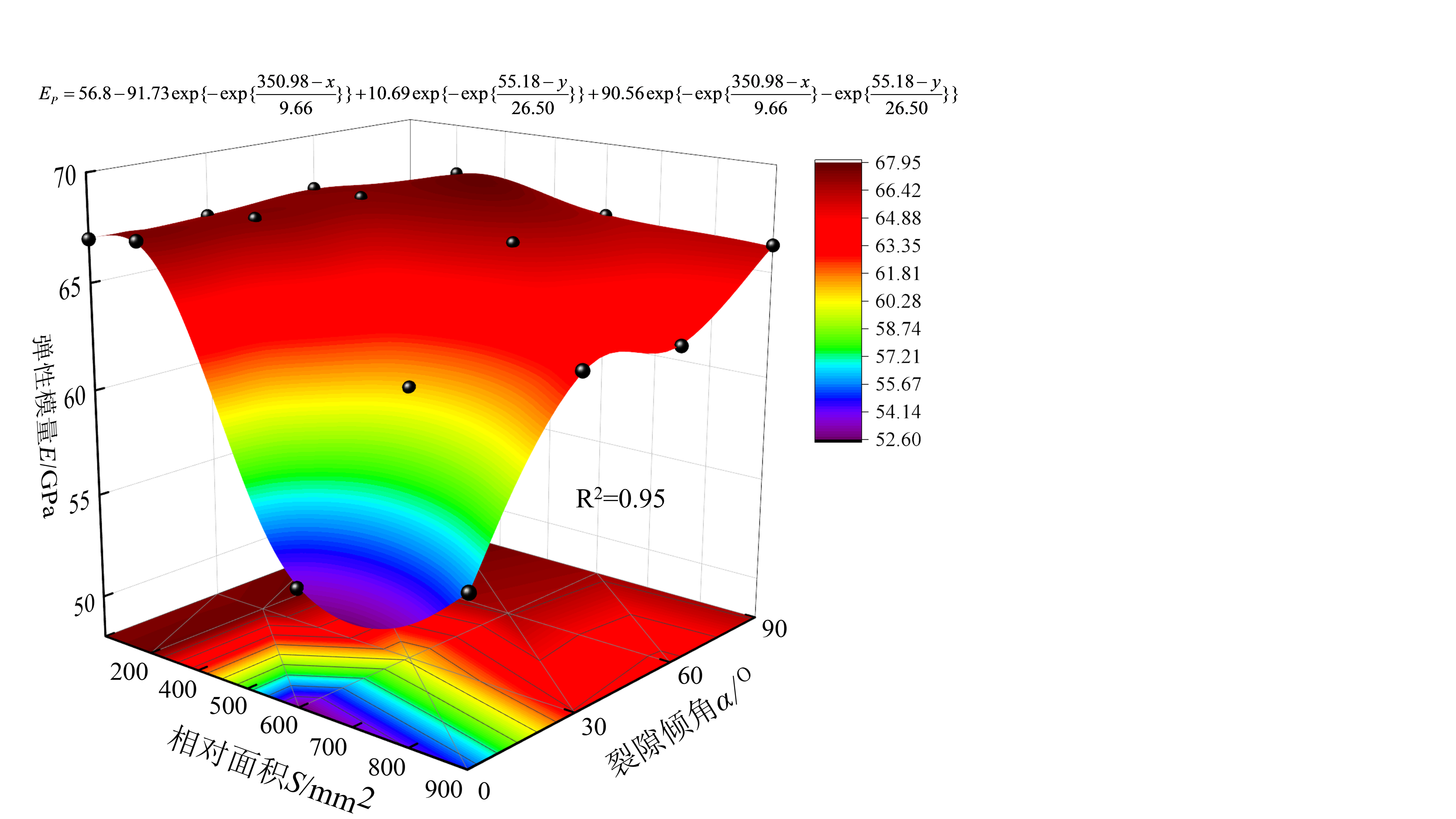

Supplement: S1 Dataset — (ZIP) [file pone.0316124.s001.zip › S1 Dataset/Fig 5. (b) Elastic modulus of precast crack inclination rock-concrete composite with different relative areas.tif]

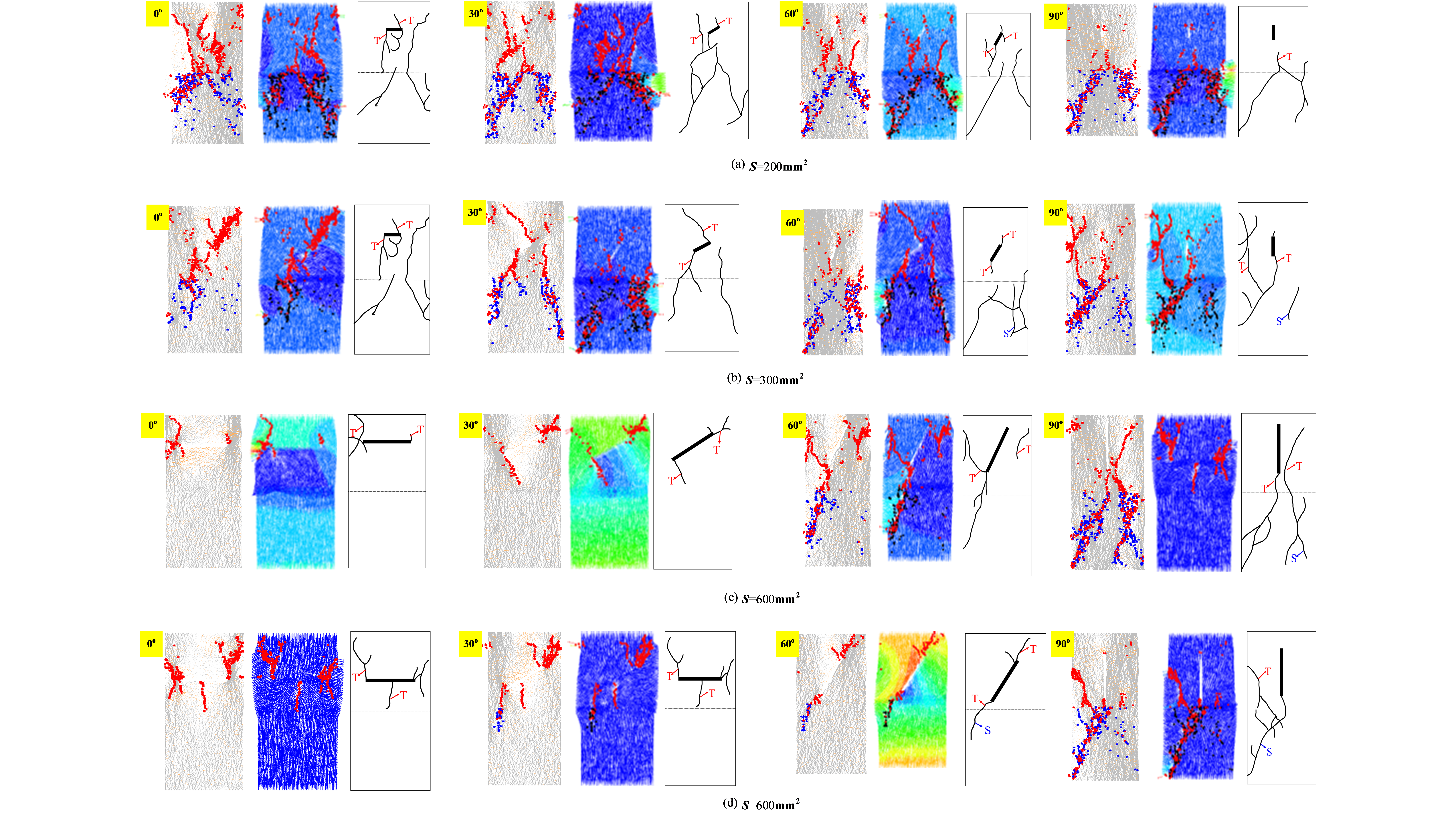

Supplement: S1 Dataset — (ZIP) [file pone.0316124.s001.zip › S1 Dataset/Fig 6. Failure modes of the precast crack inclination rock-concrete composite with different relative areas..tif]

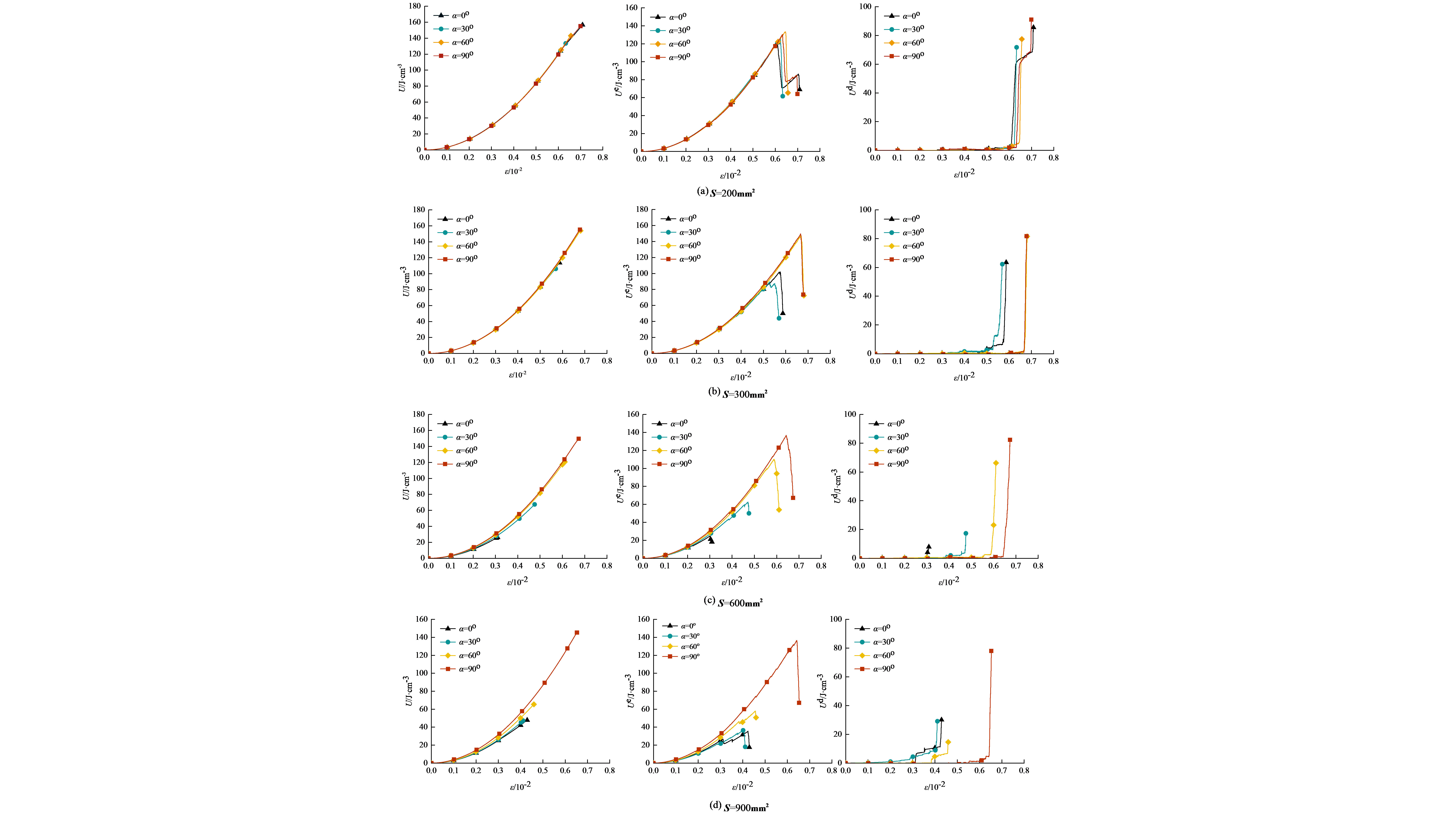

Supplement: S1 Dataset — (ZIP) [file pone.0316124.s001.zip › S1 Dataset/Fig 8. Energy evolution of granite-concrete composite with precast crack Angle under different relative areas..tif]

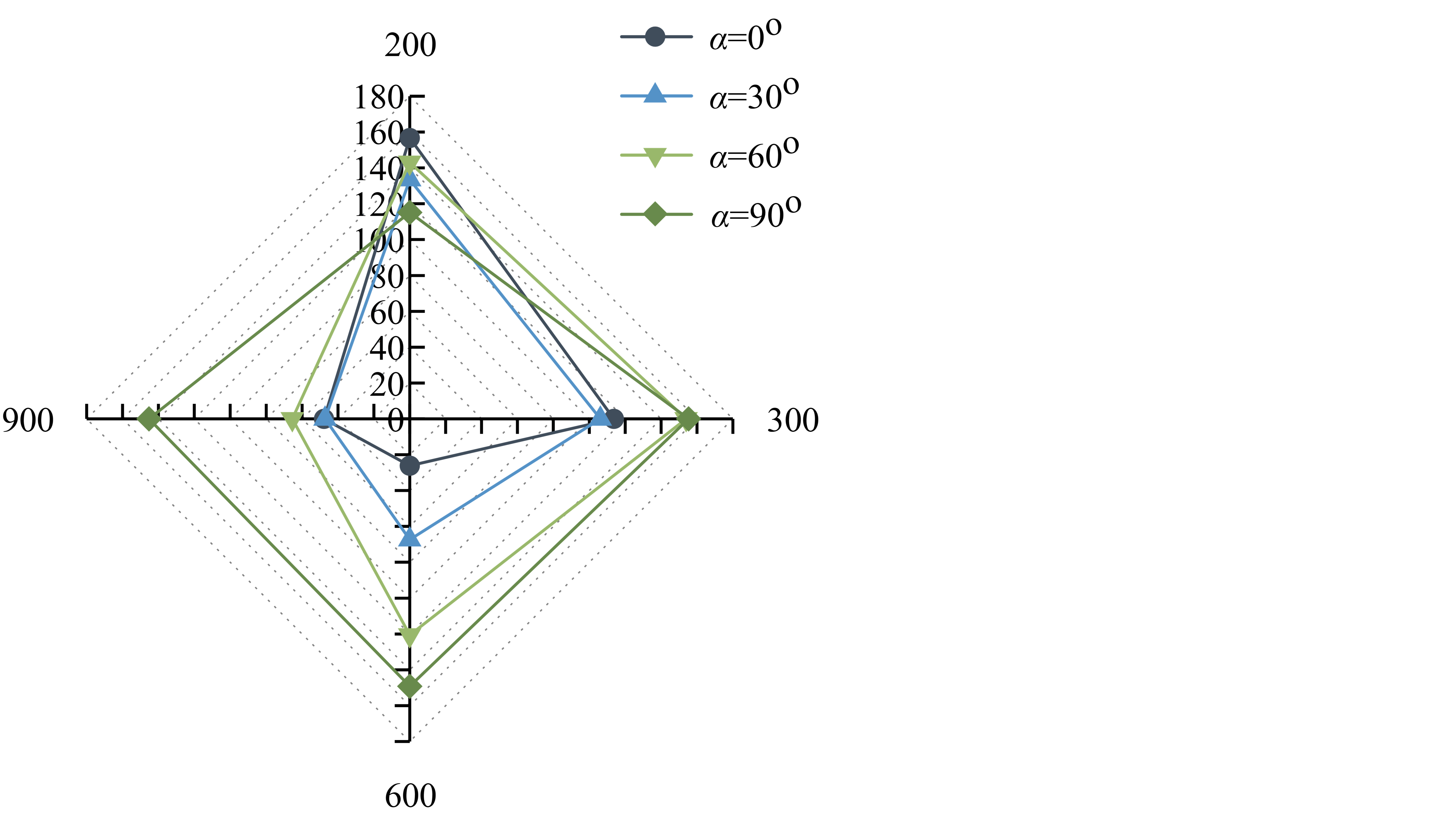

Supplement: S1 Dataset — (ZIP) [file pone.0316124.s001.zip › S1 Dataset/Fig 9. Relative area to total energy at peak point of precast fissure inclined rock-concrete composites.tif]

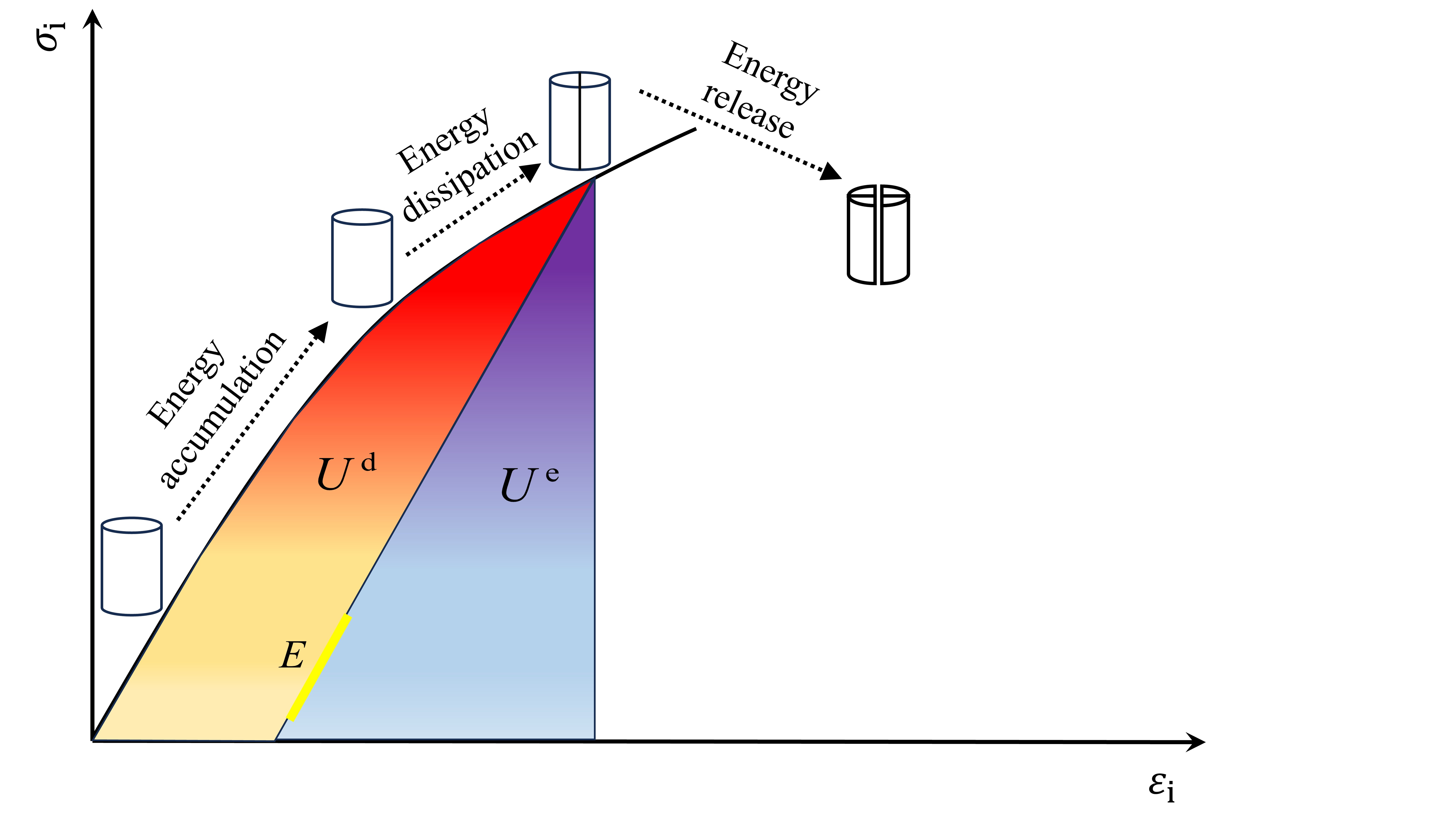

Supplement: S1 Dataset — (ZIP) [file pone.0316124.s001.zip › S1 Dataset/Fig7. Principle of energy calculation.tif]

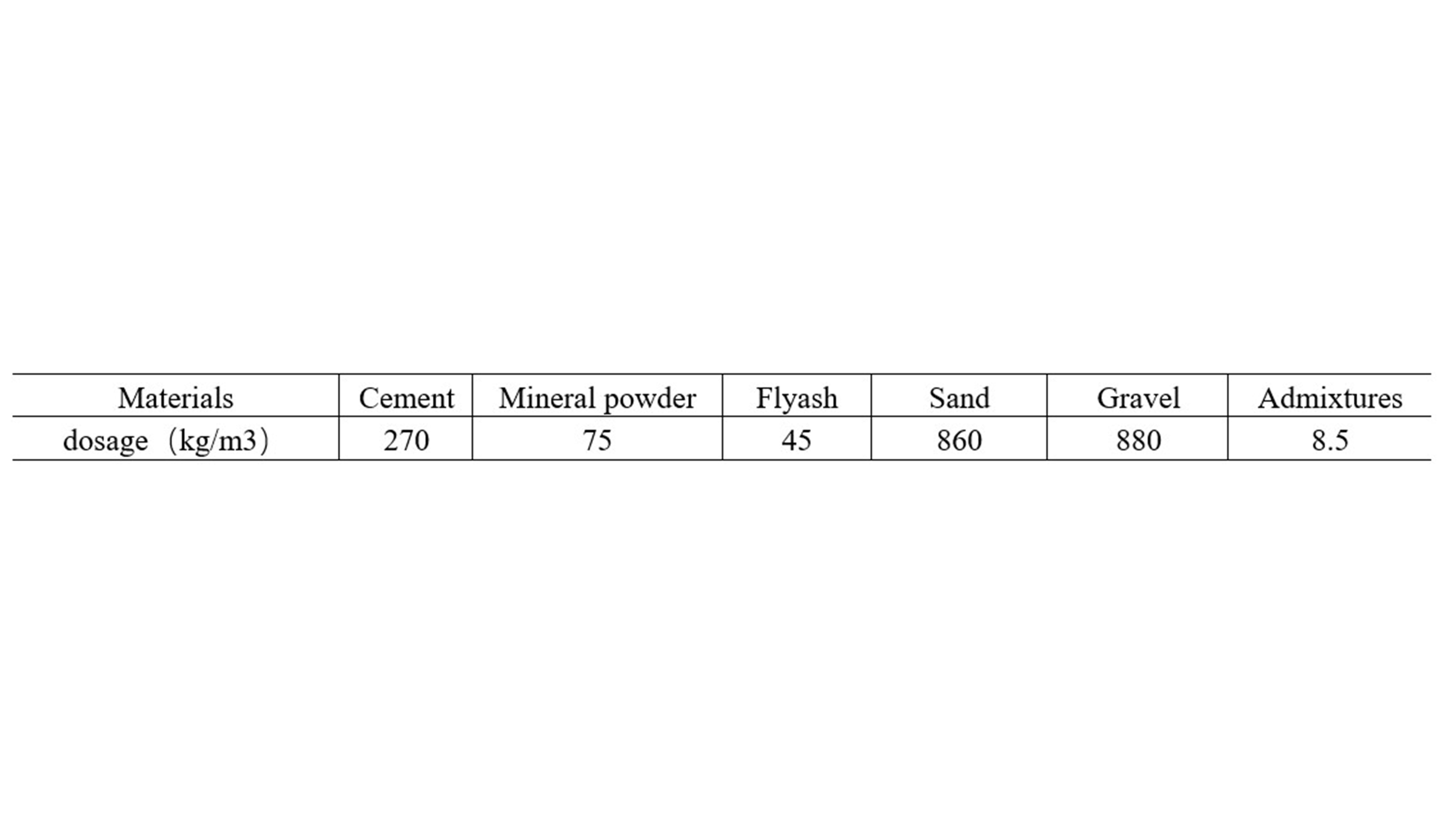

Supplement: S1 Table — (ZIP) [file pone.0316124.s002.zip › S2 Table/Table 1 Concrete mix proportion.tif]

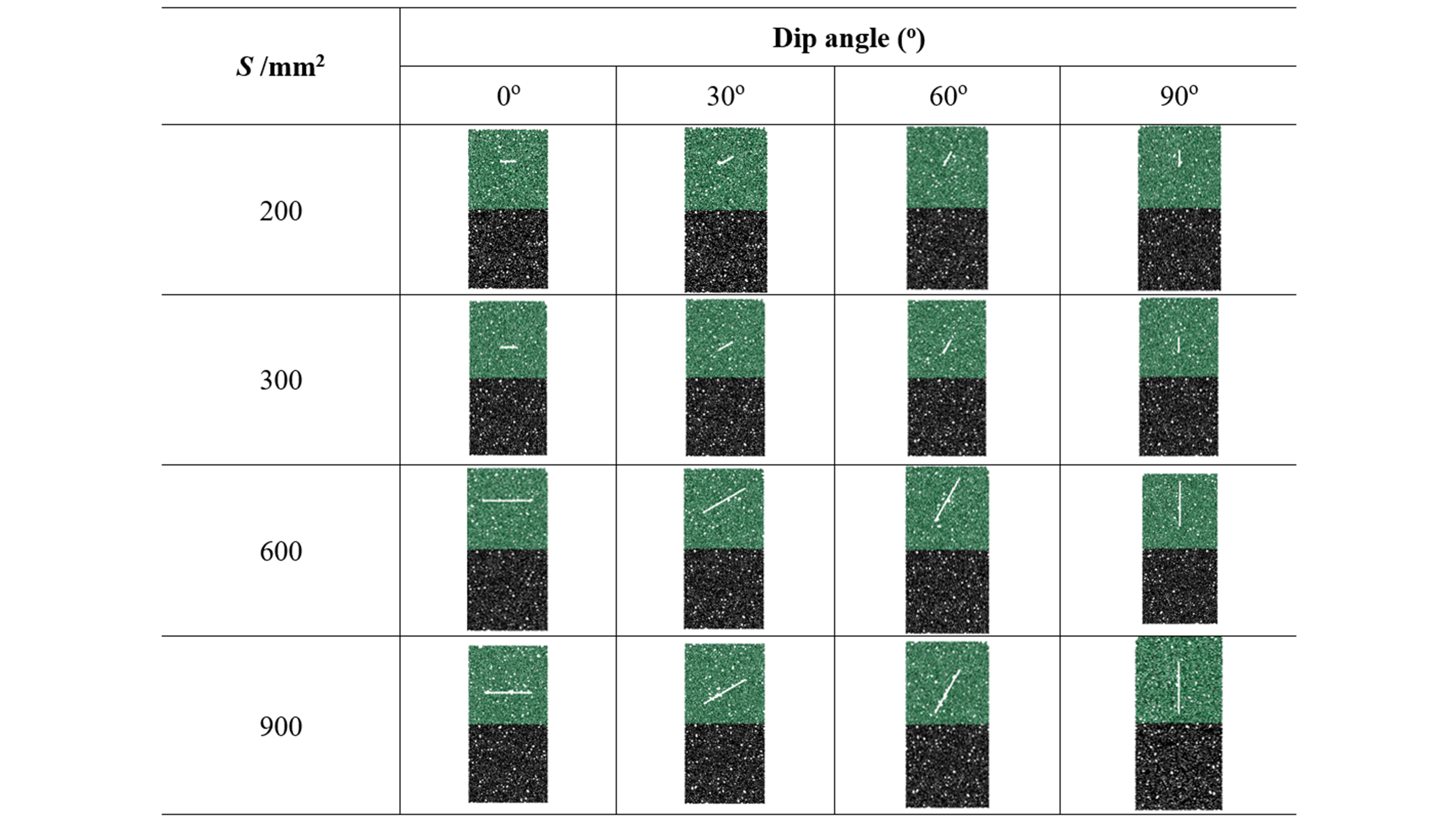

Supplement: S1 Table — (ZIP) [file pone.0316124.s002.zip › S2 Table/Table 2. PFC Simulation Mesoscopic Parameters..tif]

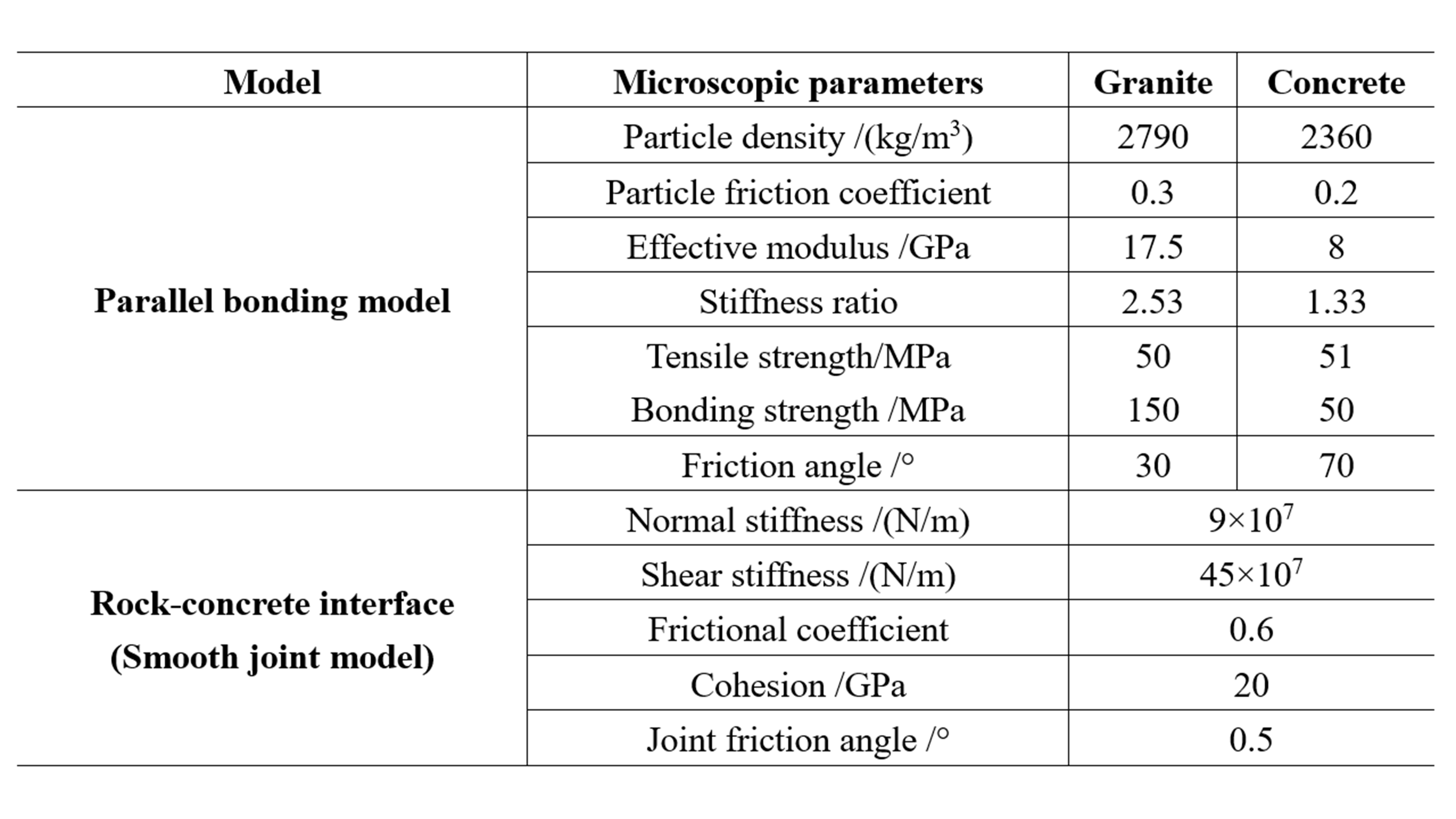

Supplement: S1 Table — (ZIP) [file pone.0316124.s002.zip › S2 Table/Table 3. Parameters PFC simulation microscopic..tif]

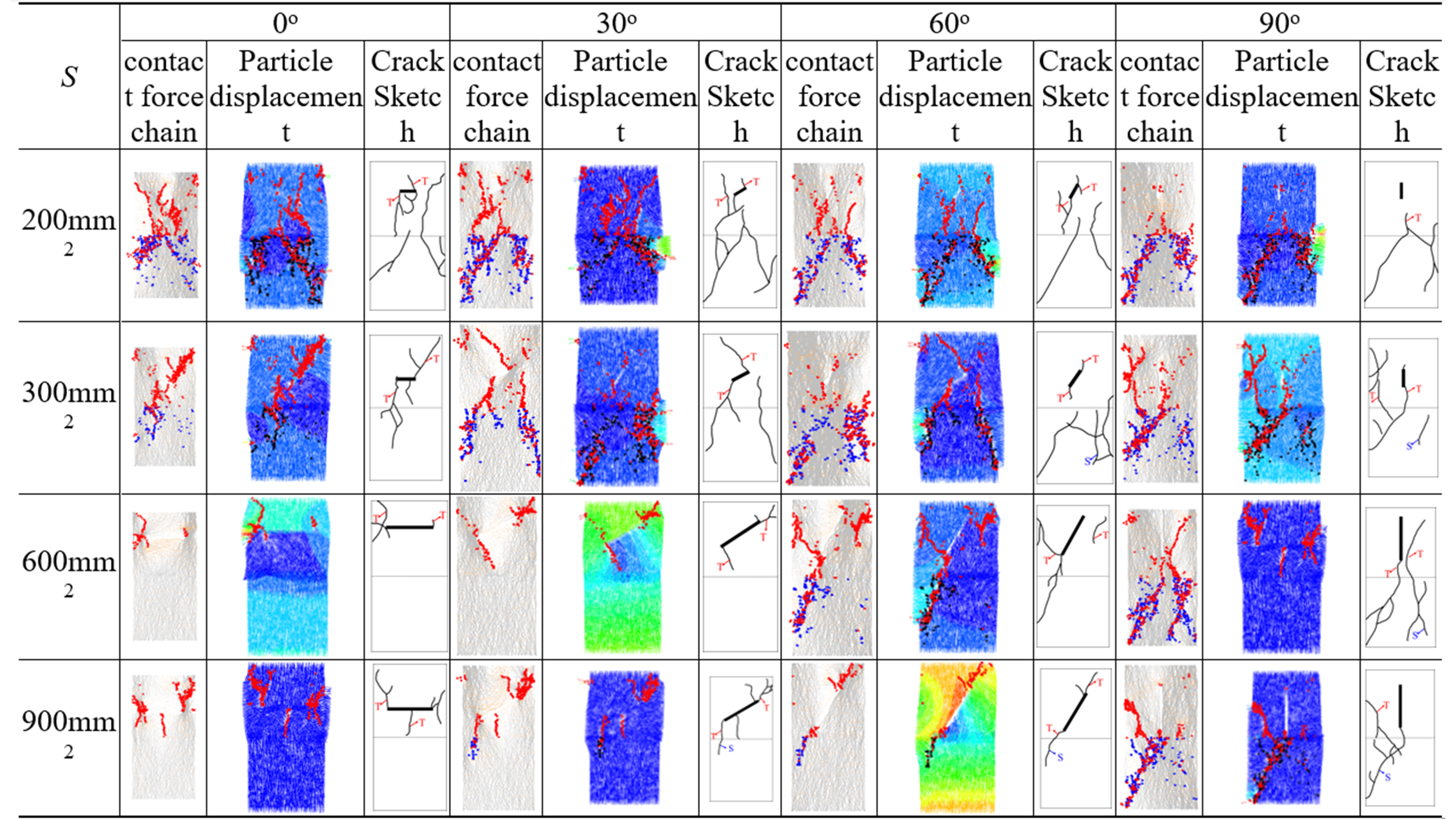

Supplement: S1 Table — (ZIP) [file pone.0316124.s002.zip › S2 Table/Table 4. Failure modes of the precast crack.tif]

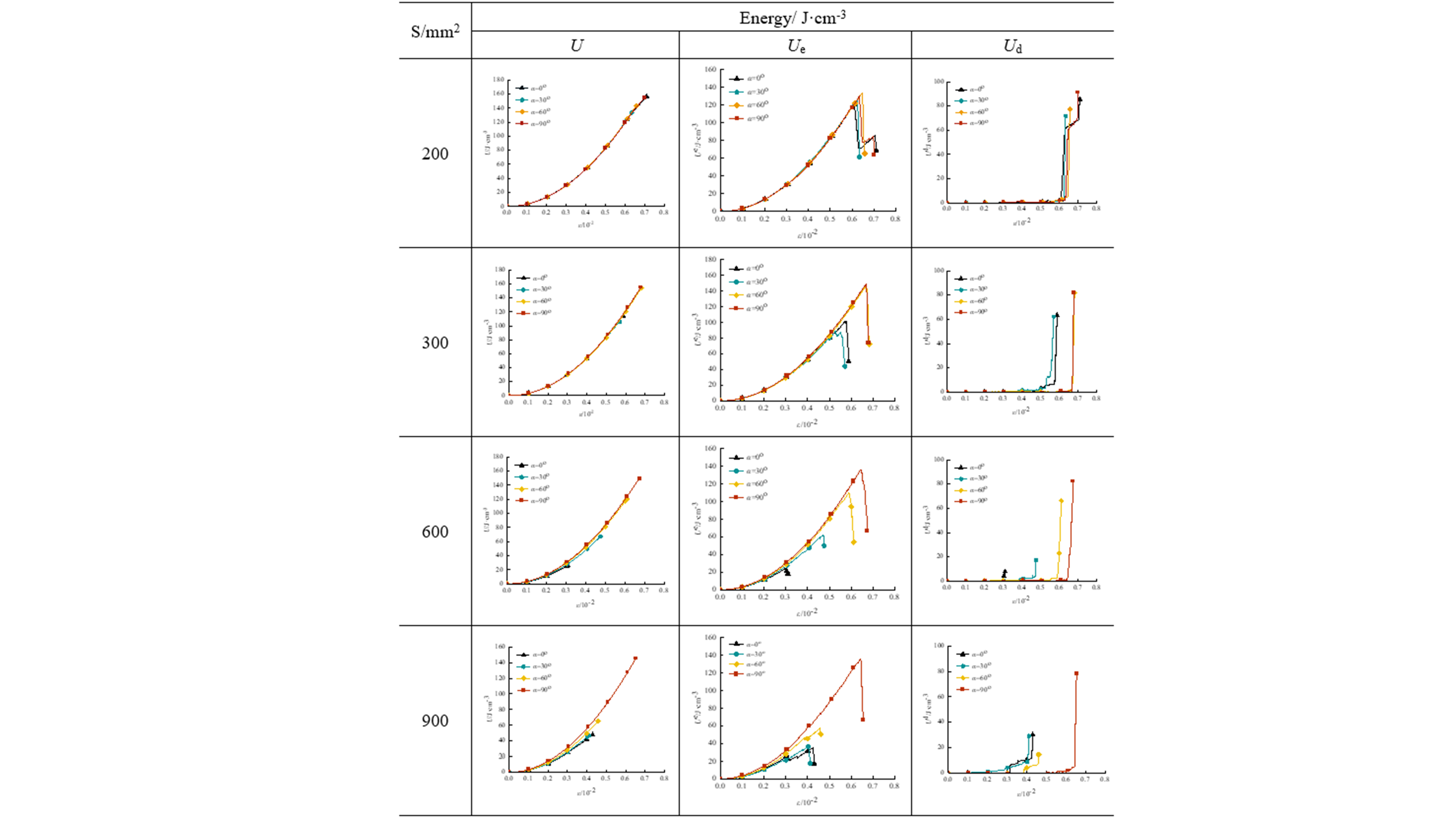

Supplement: S1 Table — (ZIP) [file pone.0316124.s002.zip › S2 Table/Table 5. Energy evolution of granite-concrete composite.tif]

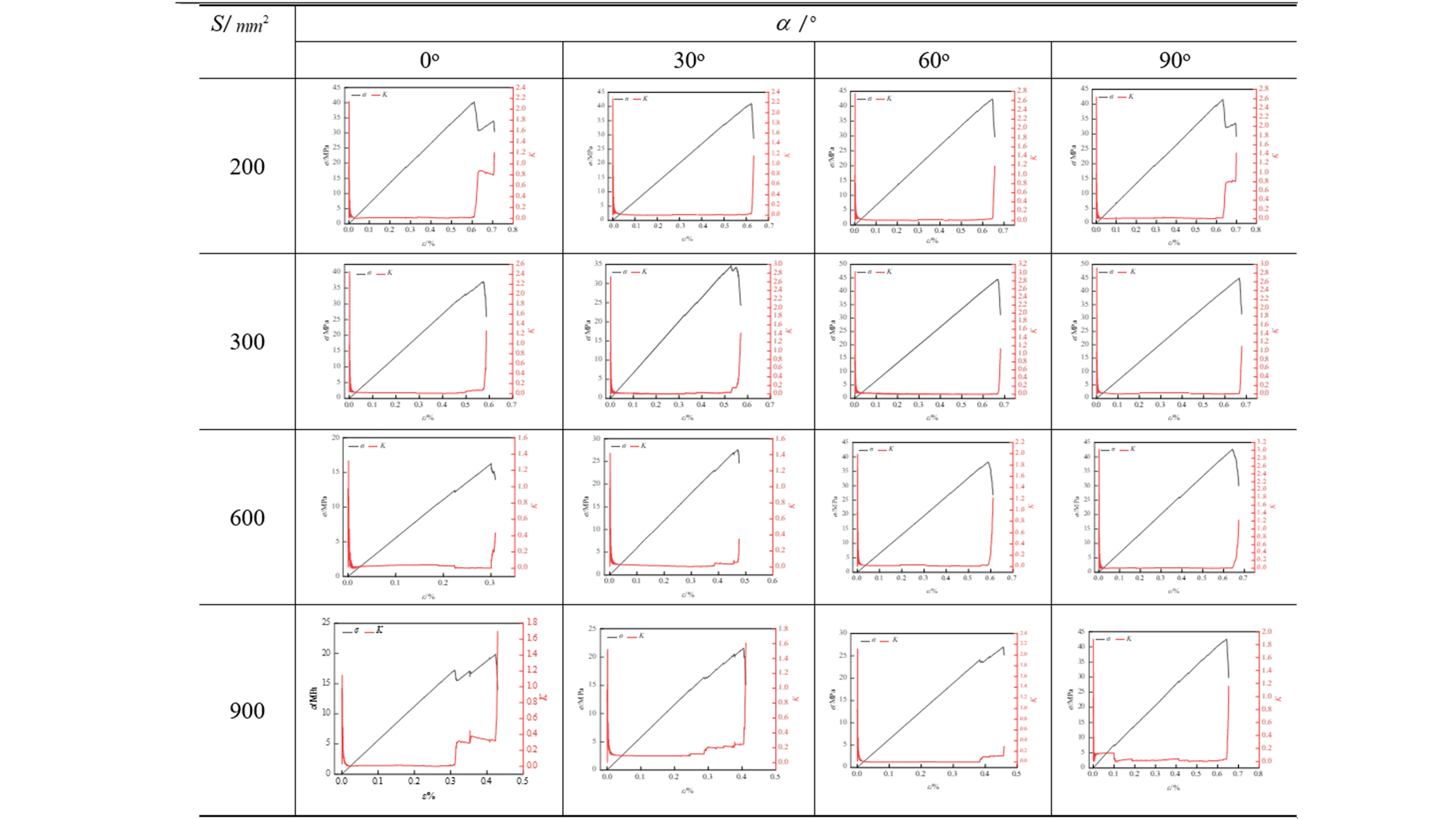

Supplement: S1 Table — (ZIP) [file pone.0316124.s002.zip › S2 Table/Table 6. Elastic energy consumption rate-stress-strain curve.tif]
